# Janus Zn-Mo nanozymes for leveraged trienzymatic anticancer therapy

**DOI:** 10.1016/j.mtbio.2025.102662

**Published:** 2025-12-09

**Authors:** Shuang Liu, Jiawei Qu, Jiating Xu, Qiang Wang, Le Zhang, Xinyu Zhang, Chunsheng Li, Yong Lu, Yi Zhong, Linbo Li, Piaoping Yang

**Affiliations:** a Key Laboratory of Forest Plant Ecology, Ministry of Education, College of Chemistry, Chemical Engineering and Resource Utilization, Northeast Forestry University, Harbin, 150040, PR China; b Heilongjiang Provincial Key Laboratory of Ecological Utilization of Forestry-Based Active Substances, Northeast Forestry University, Harbin, 150040, PR China; c Faculty of Materials Science and Energy Engineering/Institute of Technology for Carbon Neutrality, Shenzhen Institute of Advanced Technology, Chinese Academy of Sciences, Shenzhen, 518055, PR China; d Key Laboratory of Superlight Materials and Surface Technology, Ministry of Education, College of Material Sciences and Chemical Engineering, Harbin Engineering University, Harbin, 150001, PR China

**Keywords:** Catalytic anticancer, Dual-atomic nanozymes, Janus coordination, NIR-II excitation, Synergistic effect

## Abstract

Dual-atom nanozymes (DAs) have garnered immense attention for cancer treatment due to their flexible active sites and synergistic interatomic interactions. However, precisely customizing different anionic ligands for the two active centers to disrupt their local symmetry and optimize the adsorption/desorption equilibrium of intermediates remains a major challenge. Here, we report the synthesis of Janus Zn-Mo carbon-based dual-atom nanozymes (Janus Zn-Mo DAs) bio-nanoplatform featuring Zn-N_4_ and Mo-N_2_O_2_ motifs using a simple strategy. The Zn and Mo atoms in Janus Zn-Mo DAs are bridged by two nitrogen atoms, giving rise to an asymmetric structure that exhibits remarkable enzyme-mimicking properties. Both experimental and theoretical results revealed that the enhanced enzymatic behavior was driven by the asymmetric geometry of the diatomic sites, which shifted the d-band center of the active sites closer to the Fermi level, thereby facilitating electron transfer during the reaction. The enzyme-like catalytic activity of the Janus Zn-Mo DAs, coupled with their photothermal effect (*η* = 47.2 %), enables effective tumor growth inhibition *in vivo*, with an inhibition rate of up to 95 %. These findings provide crucial insights into the atomic- and electronic-level mechanisms of DAs with varying coordination environments and further promote the deep integration of nanotechnology and biology.

## Highlights

• Janus Zn-Mo carbon-based dual-atom nanozymes feature Zn-N_4_ and Mo-N_2_O_2_ motifs through a simple strategy.
• Electron interaction between Zn and Mo optimize the adsorption-desorption equilibrium of reaction intermediates.
• The synergistic effect of multiple enzyme-mimicking properties for Janus HZn-Mo DAs could effectively inhibit tumor growth.
• The obtained Janus Zn-Mo DAs exhibited high photothermal conversion efficiency (47.2 %) under NIR-II laser irradiation.

## Graphical abstract

Carbon-based Janus Zn-Mo dual-atom nanozymes with Zn-N_4_ and Mo-N_2_O_2_ moieties (Janus Zn-Mo DAs) were developed for anticancer study through optimizing the adsorption/desorption of O-containing reaction intermediates during the POD, CAT and OXD-like enzyme process. The enhanced multi-enzyme catalytic activities are attributed to the asymmetric configuration and synergistic effects of the Zn-N_4_ and Mo-N_2_O_2_ moieties.

**Figure:**
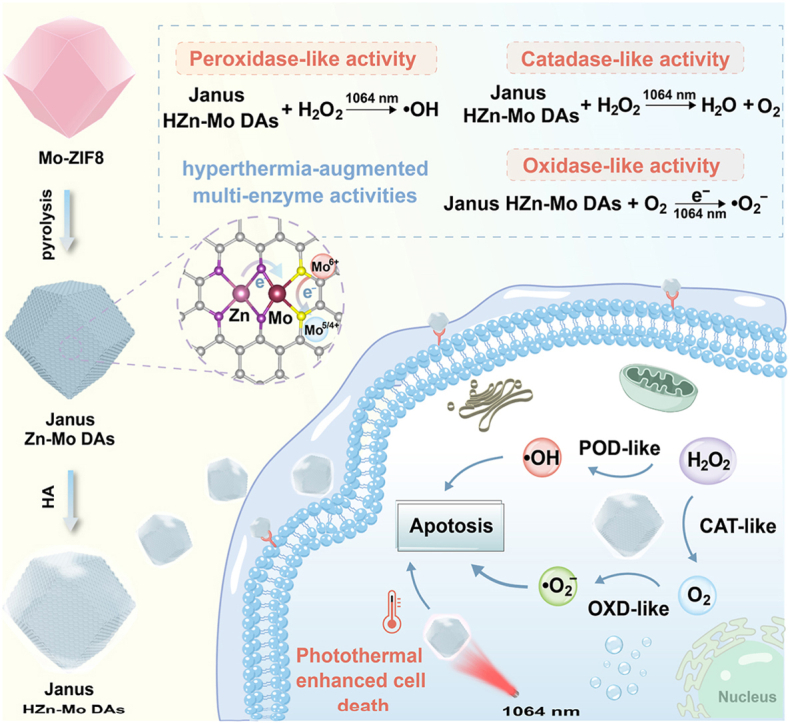

## 1 Introduction

Imidazole-based metal-organic-framework-derived single atoms nanozymes (SAs) usually have M-N_4_-C structure analogous to that of bio-porphyrins, thereby garnering considerable attention in the field of tumor catalytic therapy [[1], [2], [3], [4], [5], [6]]. However, the M − N_4_ moieties with high symmetrical electronic density distribution and single active metal atoms are unfavorable for both adsorption/desorption and activation of O-containing intermediates, thus leading to sluggish kinetic activity [7,8], It has been demonstrated that optimizing coordination environments of metal centers could effectively improve catalytic activities [[9], [10], [11], [12]]. The interaction between atomic metal centers and their substrate, as well as heteroatoms, has attracted wide attention [[13], [14], [15], [16]]. For instance, doping hetero-atoms into the carbon substrate with M−N_4_ moieties [17] can establish immediate interactions between metals and heteroatoms [18]. Specifically, at the original M − N_4_ sites, heteroatoms such as sulfur [[19], [20], [21]], phosphorus [22,23], carbon [24], or the metal center may bind ligands like OH [25], Cl [26], Br and I that may partially replace some nitrogen atoms, which directly modulates the chemical environment around the metal atoms causing the asymmetric atomic interface. The change of coordination environment can adjust the electronic structure of the active metal center, so as to effectively regulate the adsorption energy of the intermediate and improve the tumor nanocatalytic performance [27].

Actually, dual-atom nanozymes (DAs) perfectly inherit the advantages of SAs, and the binary combination of atomically dispersed active sites provides enhanced structural and compositional flexibility, synergistic effects, and novel catalytic mechanisms [[28], [29], [30]]. In detail, incorporating dual atomic sites introduces multiple top or bridge active sites, diverse absorption configurations for substrate or reaction intermediate, and synergies in the electronic structure due to metal-metal cooperativity. This can lead to novel scaling relationships in reaction intermediates, potentially breaking traditional limitations and significantly improving intrinsic catalytic efficiency [31,32]. At present, the research on DAs mainly focuses on two aspects: homonuclear [33] and heteronuclear DAs [34]. Homonuclear DAs consist of the same type of metal forming pairs of atomically dispersed active sites, while heteronuclear DAs are composed of diverse metals forming bimetallic pairs. The homonuclear DAs display unique electronic states and configurations, which can achieve high atomic loading and high catalytic efficiency [35].

Compared to homogeneous DAs, heterogeneous DAs use two or more different types of metal atoms, enabling functional complementarity and synergistic effects [36,37]. The electronic interactions between these adjacent heterometals can regulate the energy barriers of reaction intermediates species [38]. In particular, the interaction between the two metals may also optimize O-containing species adsorption/desorption and facilitate the transfer of proton-electron among reaction intermediates, which provides more favorable catalytic sites and endows DAs with multi-functional properties [39]. For instance, single Fe-N_4_ sites show a high dissociation energy of H_2_O_2_, which hinders the weakening of O=O bonds and limits the optimization of the adsorption energy of O-containing intermediates species. Notably, the introduction of neighboring Co sites can optimize the electronic structure of the single Fe-N_4_ sites and regulate the d-band center of metal sites, thus making the reaction process easier [40].

Nevertheless, recently reported DAs predominantly feature a single coordinating anion, as it proves challenging to precisely manage the separate coordination of two distinct metal atoms [41,42]. To further enhance the catalytic efficiency of DAs in catalytic reaction processes, a crucial but challenging strategy involves tailoring distinct anionic ligands for the two metals to form a Janus dual-metal site. This approach aims to maximize functionality by multiple synergistic effects. However, due to the absence of effective synthesis methods and the difficulty in distinguishing and identifying the exact amorphous structure of complex active sites, Janus-type DAs with precisely defined multiple ligands have rarely been reported [43]. Employing effective strategies to precisely modulate the coordination centers of nanozymes to overcome the poor adsorption/activation properties of H_2_O_2_ or O_2_ during enzymatic reactions is highly desirable for efficient tumor catalytic therapy. Therefore, the strategic design of efficient nanozymes that can more effectively simulate the active sites and spatial configurations of enzymes for tumor catalytic therapy remains challenging [[44], [45], [46], [47]].

**Scheme 1:**
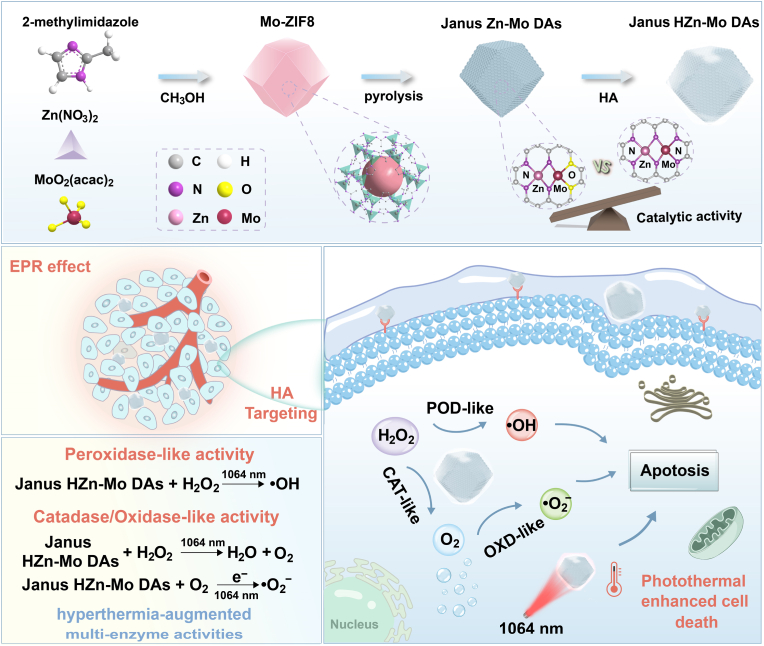
The schematic illustration for the preparation process and the theranostic mechanism of the Janus HZn-Mo DAs.

Here, we construct a general and simple strategy for synthesizing Janus Zn-Mo dual atomic sites embedded in N/O-doped graphitic carbon (Janus Zn-Mo DAs) through *in situ* encapsulation of Zn/Mo binuclear sites into zeolitic imidazolate frameworks (ZIF-8) followed by carbonization (Scheme 1). The synthesized Janus DAs contained adjacent ZnN_4_ and MoN_2_O_2_ moieties in a heterogeneous geometric configuration. Combining the experimental results and the density functional theory (DFT) calculations, possible structures for Janus Zn-Mo DAs were postulated. The electron interaction between Zn and Mo tuned position of the d-band center of the active sites and balanced the adsorption-desorption of reaction intermediates. To improve the targeting capacity *in vivo*, the surface of Janus Zn-Mo DAs was further modified with hyaluronic acid (Janus HZn-Mo DAs). Janus HZn-Mo DAs could function as peroxidase (POD)-mimic to catalyze the decomposition of cellular endogenous H_2_O_2_ into a highly cytotoxic hydroxyl radical (•OH). Meanwhile, Janus HZn-Mo DAs not only serve as a catalase (CAT)-mimic to catalyze the decomposition of cellular endogenous H_2_O_2_ to produce O_2_ to relieve hypoxia, but also serve as an oxidase (OXD)-mimic to continuously catalyze the conversion of O_2_ to highly cytotoxic •O_2_^−^ radicals. The *in vitro* and *in vivo* experiments results showed that Janus HZn-Mo DAs could effectively inhibit tumor growth through synergistic therapeutic effects, combining photothermal-augmented nanocatalytic therapy with ideal tumor targeting effect.

## 2 Materials and methods section

### 2.1 Preparation of Mo-ZIF8 precursors

First, 2-methylimidazole (3.7 g) was dissolved in 80 mL methanol with sonification for 30 min to form a homogeneous solution in flask A; Zn(NO_3_)_2_·6H_2_O (1.67 g) and MoO_2_(acac)_2_ (92 mg) was dissolved in 40 mL methanol under ultrasound for 15 min to form a clear solution in flask B. Then, the mixture in flask B was dropwise added into flask A with vigorous stirring for 12 h at room temperature. The obtained product was separated by centrifugation and washed subsequently with methanol for three times and finally dried overnight at 60 °C under vacuum.

### 2.2 Preparation of Janus Zn-Mo DAs

The precursors of Mo-ZIF-8 were pyrolyzed in an aluminum oxide ceramic boat in a tube furnace. The temperature was increased to 800 °C with a heating rate of 5 °C min^−1^ for 3 h under flowing nitrogen gas and then naturally cooled down to the room temperature.

### 2.3 Preparation of HA-modified Janus Zn-Mo DAs (designated as Janus HZn-Mo DAs)

Briefly, 50 mg of Zn-Mo DAs was first dissolved in 25 mL of H_2_O, and 50 mg of HA was dispersed in another 25 mL H_2_O. These two solutions were mixed together and shaken for 3 h. Then, the homogeneous solution was centrifuged, and the unreacted molecules were removed by rinsing several times. Finally, the resultant samples were dried overnight at 60 °C under vacuum for subsequent use.

### 2.4 Detection of ROS by ESR

The generation of •OH and •O_2_^−^ were further measured by the electron spin resonance (ESR) spectrometry, in which TEMP and DMPO were employed as a spin-trap agent for •O_2_^−^ and •OH, respectively. The detailed experimental procedures were as follows: for •OH generation, 1 mg mL^−1^ ZIF-8, Zn SAs and Janus Zn-Mo DAs was added into pH 6.5 PBS containing 10 mM H_2_O_2_ at 37 °C. After incubation for the same times, 5 mM spin-trap agent was added. Then, the signal was immediately determined by an ESR spectrometer.

### 2.5 *In vitro* photothermal effect of Janus Zn-Mo DAs

In the first experimental group, to obtain different concentrations of 62.5, 125, 250, 500 and 1000 μg mL^−1^, a certain amount of Janus Zn-Mo DAs was dispersed in distilled water. Subsequently, the suspension was irradiated (1064 nm, 1.0 W cm^−2^) for 10 min. In the second experimental group, the Janus Zn-Mo NPs solution (1 mg mL^−1^) was irradiated under various power densities (0.8, 1.0 and 1.2 W cm^−2^). Finally, photothermal stability of Janus Zn-Mo DAs solution was investigated. The temperature variations of system were monitored by IR thermal imager (TI100 FLKTI100).

### 2.6 Cellular uptake of Janus HZn-Mo DAs

First, ethanol solutions (20 mL) containing FITC (2 mg mL^−1^) were stirred vigorously together with 10 mg of Janus HZn-Mo DAs for 24 h to synthesize FITC-modified Janus HZn-Mo DAs. Afterward, to obtain monolayer HeLa cells, they were seeded in a six-well plate and cultured overnight. Then, Janus HZn-Mo DAs with FITC decoration (1 mL, 0.5 mg mL^−1^) was transferred to the six-well plates and further incubated for 1 h. After the cells were washed with PBS, DAPI as a nuclear stain was added to each well to stain the cells for 10 min. Further, 1 mL of glutaraldehyde with a concentration of 2.5 % was added to fix the cells for 10 min; then the wells were rinsed with PBS several times. Finally, the fluorescence images were recorded by confocal laser scanning microscope (CLSM).

## 3 Results and discussion

### 3.1 Construction and characterization of Janus Zn-Mo DAs

**Fig. 1:**
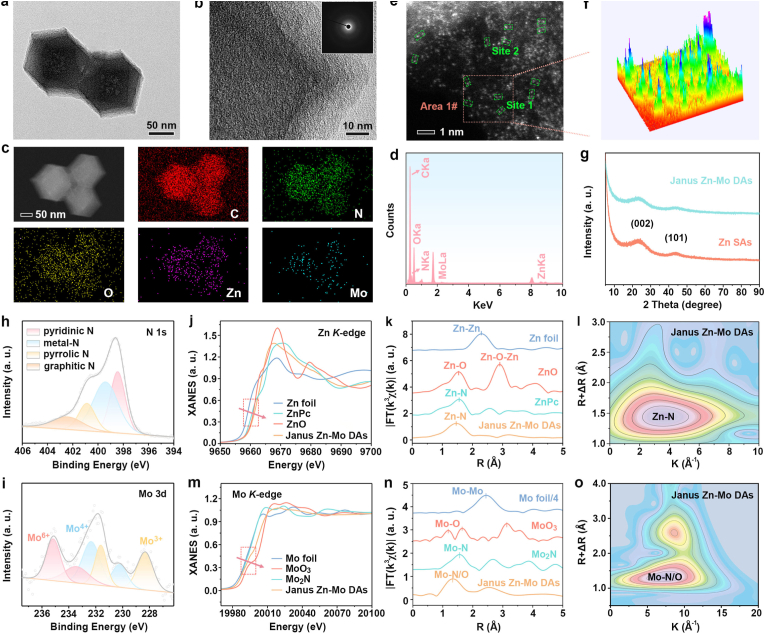
TEM image of Janus Zn-Mo DAs (a). High-resolution TEM image of the Janus Zn-Mo DAs (Inset: SAED pattern of Janus Zn-Mo DAs) (b). Elemental mapping of Janus Zn-Mo DAs (c). EDS spectrum of Janus Zn-Mo DAs (d). Aberration-corrected HAADF-STEM of Janus Zn-Mo DAs (e). 3D pseudo-color surface plot of the Area 1#) (f). XRD patterns of Zn SAs and Janus Zn-Mo DAs (g). The high-resolution N 1s (h) and Mo 3d (i) XPS spectra of Janus Zn-Mo DAs. Zn K-edge XANES spectra (j). Fourier transform k^2^-weighted EXAFS spectra of Janus Zn-Mo DAs and the reference samples of Zn foil, Zn *Pc* and ZnO at Zn *K*-edge (k). Wavelet transform for the k^2^-weighted Zn *K*-edge EXAFS signals of Janus Zn-Mo DAs (l). Mo *K*-edge XANES spectra (m). Fourier transform k^2^-weighted EXAFS spectra of Janus Zn-Mo DAs and the reference samples at Mo *K*-edge (n). Wavelet transform for the k^2^-weighted Mo *K*-edge EXAFS signals of Janus Zn-Mo DAs (o).

The preparation of Janus Zn and Mo dual-atom nanozymes is illustrated in Scheme 1. MoO_2_(acac)_2_ served as both the Mo and O source. Mo-doped ZIF-8 (Mo-ZIF8) was constructed in a one-step process and then pyrolyzed under a nitrogen atmosphere to immobilize Zn and Mo dual single atoms on N/O-doped carbon framework, denoted as Janus Zn-Mo DAs. Scanning electron microscopy (SEM) and transmission electron microscopy (TEM) images revealed that the ZIF-8 nanocrystals exhibited a uniform rhombic dodecahedron morphology (Fig. S1a–b). Subsequently, ZIF-8 was utilized as the host for Mo doping. As shown in Fig. S1c–d, the rhombic dodecahedron morphology of Mo-ZIF8 was preserved, however, the nanoparticle size was slightly larger than that of pristine ZIF-8. The X-ray diffraction (XRD) was used to investigate the structure of the prepared nanozymes. As shown in Fig. S2, the characteristic peaks of Mo-ZIF8 and ZIF-8 were preserved with very little change and matched well with the simulated results, demonstrating that the framework structure of ZIF-8 was not destroyed by Mo doping. The Brunauer-Emmett-Teller (BET) specific surface areas and average pore diameter of ZIF-8 were observed to be 1280.3 m^2^ g^−1^ and 1.21 cm^3^ g^−1^, respectively (Fig. S3). Janus Zn-Mo DAs were then fabricated by pyrolyzing Mo-ZIF8 nanoparticles and Zn and Mo atoms were anchored on a porous N/O-doped carbon support. Specifically, the molecular fencing role of the ZIF-8 precursor leads to the generation of a porous nitrogen-doped carbon framework intermediate, which provides abundant coordination sites (such as N and O) for anchoring Zn and Mo species. After pyrolysis, a slight roughness was observed on the porous-Janus Zn-Mo DAs (Fig. 1a). High-resolution TEM (HR-TEM) image and selected area electron diffraction (SAED) results revealed the amorphous structure of the Janus Zn-Mo DAs without the formation of small metallic Zn/Mo-based nanoparticles (Fig. 1b). Additionally, elemental mapping and energy-dispersive spectrometry (EDS) analyses (Fig. 1c and d) showed that C, N, O, Zn, and Mo were uniformly distributed in the architecture of the Janus Zn-Mo DAs. The loading amount of Zn and Mo dual single atoms was determined approximately 10.22 and 1.57 wt%, respectively, using inductively coupled plasma optical emission spectrometry (ICP-OES) (Table S1).

As shown in Fig. S4, a large surface area of 631.1 m^2^ g^−1^ of porous-Janus Zn-Mo DAs was obtained from BET measurement and the pore size distribution curve, which was beneficial to the exposure of Zn and Mo dual active species and high-efficiency mass transport for catalytic processes. Furthermore, intuitive insight into the Zn and Mo species in the Janus Zn-Mo DAs was obtained using aberration-corrected high-angle annular dark-field scanning transmission electron microscopy (HAADF-STEM). In Fig. 1e, the isolated bimetallic sites (green boxes) were clearly observed for the carbon substrate, the corresponding three-dimensional (3D) pseudo-color surface plot of Area 1# vividly showed the isolated metallic atoms (Fig. 1f). In the intensity profiles, the distances of two typical pairs were 0.22 and 0.23 nm (Fig. S5a). As shown in Fig. S5b, the histogram reveals a predominant distance distribution peak centered at approximately 0.22 nm for the Zn-Mo pairs. The XRD patterns of Zn SAs and Janus Zn-Mo DAs revealed two broad diffraction peaks corresponding to the (002) and (101) crystal facets of graphitic carbon, respectively, excluding the formation of Zn and/or Mo metal species (Fig. 1g). Raman spectra of Zn SAs and Janus Zn-Mo DAs were displayed in Fig. S6, where the D band located near 1350 cm^−1^ corresponds to disordered carbon and the G band located near 1580 cm^−1^ represents graphitic carbon [48]. The I_D_/I_G_ values of Zn SAs and Janus Zn-Mo DAs were determined to be 1.36 and 1.41, respectively, which were due to the richer topological defect sites after the doping of Mo species. Subsequently, the colloidal stability of Janus Zn-Mo DAs in three physiologically relevant environments was investigated. As shown in Fig. S7, the hydrodynamic diameter of nanozymes remained nearly constant in both PBS and acidic PBS containing GSH over 48 h. In a simulated blood environment (PBS containing 10 % FBS), an initial increase in size was observed, which is consistent with the rapid formation of a protein corona. It is worth noting that after this initial stage, the size tends to be stable and remains stable during the testing period. This non-continuous aggregation phenomenon indicates that Janus Zn-Mo DAs have good colloidal stability in complex physiological environments.

The chemical compositions of the Janus Zn-Mo DAs were investigated using X-ray photoelectron spectroscopy (XPS). As shown in Fig. S8a, in the XPS survey spectra of the ZIF-8, Zn SAs, Mo-ZIF8, and Janus Zn-Mo DAs, the presence of Zn elements was confirmed that the Zn species were successfully immobilized on the carbon carrier. The peak corresponding to Mo species was not observed because of its low content. The Zn 2p XPS spectrum of Janus Zn-Mo DAs slightly shifted toward higher binding energy relative to Zn SAs (Δ*E* = 0.45 eV), possibly because the introduction of the Mo served as electron acceptor (Fig. S8b). Some of the electrons on Zn were transferred to the Mo atoms, resulting in an increase in the electron cloud density of the Mo atoms and optimizing their corresponding 3d orbitals according to Equation (1):

(1) Mo^6+^ + e^−^ → Mo^5+^

To probe the structural integrity, we recovered the nanozymes after a 48 h incubation in the simulated tumor interstitial fluid (acidic PBS with GSH) and analyzed them by XPS. The XPS spectra revealed that the binding energies of Zn 2p and Mo 3d peaks remained virtually unchanged compared to the pristine sample. This strongly indicates that the chemical states and coordination geometry of the Zn and Mo sites remain intact in complicated environment, which is crucial for maintaining their catalytic activity (Fig. S8c–d). The ultraviolet photoelectron spectroscopy (UPS) of Zn SAs and Janus Zn-Mo DAs was measured. As shown in Fig. S8e, the work functions (*W*_F_) were calculated to be 4.57 and 4.39 eV for Zn SAs and Janus Zn-Mo DAs, respectively. These results demonstrated that Janus Zn-Mo DAs had a lower *W*_F_ after Mo doping can facilitate electron transfer. The C 1s spectrum of the Janus Zn-Mo DAs showed three deconvoluted peaks at 284.7, 285.8, and 287.2 eV, which were assigned to the C=C, C=N and C=O groups [49], respectively (Fig. S9). The high-resolution spectrum of N 1s for Janus Zn-Mo DAs displayed four types of N species, including pyridinic N at 398.4 eV, metal-N at 399.3 eV, pyrrolic N at 400.8 eV, and graphitic N at 402.3 eV [50], where pyridinic N can supply plenty of anchor points for a metal site (Fig. 1h). Collectively, these analyses preliminarily indicated that N-coordinated metal single-site moieties (metal-N) were present in the Janus Zn-Mo DAs. As depicted in Fig. 1i, the high-resolution XPS spectrum of Mo 3d consisted of six peaks representing three ionic species: 231.6 and 228.3 eV (Mo^3+^), 232.3 and 230.2 eV (Mo^4+^), and 235.1 and 233.4 eV (Mo^6+^) [51]. These results proved the successful introduction of Zn and Mo dual single atoms and the formation of metal-N species.

The coordination environment and chemical states of atomic Zn and Mo in the Janus Zn-Mo DAs were further investigated by X-ray absorption near edge structure (XANES). The XANES region offered information about the oxidation state of Zn (Fig. 1j). For Janus Zn-Mo DAs, the position of Zn *K*-edge absorption edge was slightly higher than that of ZnO, indicating that the valence state of Zn was higher than +2. The bonding environment of Zn in Janus Zn-Mo DAs was revealed by Fourier-transformed R-space curves of the Zn *K*-edge extended X-ray absorption fine structure (EXAFS) spectra. There was an intense Zn-Zn peak at around 2.3 Å in Zn foil, which disappeared in the R-space spectrum of Janus Zn-Mo DAs. This confirmed that Zn-Zn bonds were not formed, which was in agreement with the XRD result. For Janus Zn-Mo DAs, the first coordination shell peak was the Zn-N coordination at approximately 2.00 Å with a coordination number of 4.2 [52], demonstrating each Zn atom was coordinated with four N atoms (Fig. 1k and Table S2). A higher resolution wavelet transformation (WT) of Zn-edge oscillations was performed to confirm the atomically dispersed single sites in Janus Zn-Mo DAs. Compared to the Zn foil, ZnO and ZnPc contour plot, the Janus Zn-Mo DAs exhibited a maximum intensity and belonged to Zn-N coordination (Fig. S10 and Fig. 1l). The fitted EXAFS spectrum is shown in Fig. S11, which is consistent with the experimental spectrum for Janus Zn-Mo DAs.

The Mo *K*-edge absorption edge position for Janus Zn-Mo DAs was between Mo_2_N and MoO_3_, indicating that the valence state of Mo was intermediate between +1.5 and + 6 (Fig. 1m). The atomic structures of Janus Zn-Mo DAs were investigated by FT k^2^-weighted EXAFS. As shown in Fig. 1n, the FT-EXAFS spectra of Janus Zn-Mo DAs showed a primary peak at ∼1.77 Å, which is attributed to Mo–O/N coordination [53]. As shown in Fig. S12a, the fitting results indicate that Mo exhibits coordination with two N atoms and two O atoms in Janus Zn-Mo DAs. Compared to the WT contour plots of other references (Fig. S12b–d), the WT-EXAFS analysis of Janus Zn-Mo DAs was consistent with FT-EXAFS results, verifying the existence of a Mo–N/O path (Fig. 1o). The specific coordination configurations of the metal sites were determined based on the fitting results of the quantitative least-squares EXAFS curve. As shown in Fig. S13a and Table S2, the coordination numbers of the Mo-O and Mo-N paths for the Janus Zn-Mo DAs are both approximately 2.0 according to the best-fitting results. In addition, the existence of a Zn-N, Mo-O and Mo-N group and the absence of Zn-O group in the FT-IR spectrum for Janus ZnMo DAs confirmed that the Zn center was coordinated with N rather than with O, and the Mo center coordinated with both N and O (Fig. S13b).

### 3.2 In vitro evaluations of the multi-enzymatic catalytic activities of Janus Zn-Mo DAs

**Fig. 2:**
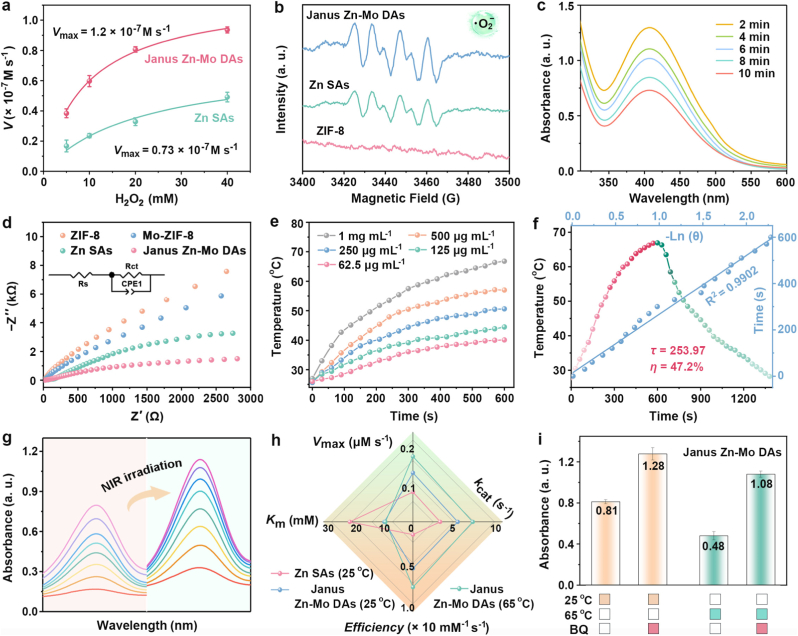
The Michaelis–Menten kinetic analysis of different nanozymes-mediated POD-like activity. Data are presented as mean ± S.D. (n = 3) (a). ESR spectra of •O_2_^−^ generation after different conditions (b). The changes in the UV–Vis absorption spectra of the mixed solution of H_2_O_2_ and Ti(SO_4_)_2_ treated with Janus Zn-Mo DAs at different times (c). Electrochemical impedance spectroscopy of ZIF-8, Mo-ZIF8, Zn SAs and Janus Zn-Mo DAs (d). Temperature elevation curves of Janus Zn-Mo DAs with various concentrations under NIR-II laser irradiation (1064 nm, 1.0 W cm^−2^) for 10 min (e). Temperature change curve and time constant of Janus Zn-Mo DAs for calculation of the photothermal conversion efficiency (f). UV–Vis absorption spectra of Janus Zn-Mo DAs with H_2_O_2_/TMB in the absence or presence of NIR irradiation (g). Comparison of POD-like kinetics for Zn SAs (25 °C), Janus Zn-Mo DAs (25 °C), and Janus Zn-Mo DAs (65 °C) (h). The absorbance changes of MB aqueous solutions under different conditions. Data were presented as mean ± S.D. (n = 3) (i).

Initially, the POD-mimic catalytic activities of Janus Zn-Mo DAs were evaluated by a typical colorimetric analysis based on 3,3′,5,5′-tetramethyl-benzidine (TMB). The Janus Zn-Mo DAs exhibited a much higher catalytic performance than the Zn SAs (Fig. S14a), proving that the dual active site center had better catalytic performance. Moreover, the catalytic activity after 48 h in the simulated tumor interstitial fluid (acidic PBS with GSH) showed no significant decrease compared to the fresh nanozymes, confirming that the catalytic activity was well preserved (Fig. S14b). In Fig. S14c, XRD patterns of Janus Zn-Mo DAs disclosed that the structure of Janus Zn-Mo DAs had no obvious change after three catalytic cycles. Notably, there is a negligible decrease in absorbance of oxTMB after three catalytic cycles, indicating that Janus Zn-Mo DAs have good catalytic stability (Fig. S14d). To confirm that the structure remains intact after use, we recovered the nanozymes following the catalytic reaction. High-resolution XPS analyses of the recovered nanozymes showed patterns nearly identical to those of the as-synthesized sample, indicating good structural integrity was maintained even after catalytic application (Fig. S14e–f). The catalytic efficiency of Zn SAs and Janus Zn-Mo DAs was evaluated by Michaelis-Menten steady-state kinetics according to Equations (2), (3), (4). The catalytic reaction kinetics were quantified by the addition of nanozymes and TMB under mildly acidic conditions, using different concentrations of H_2_O_2_ solution (5, 10, 20 and 40 mM) as the substrate. As shown in Fig. S15, the Michaelis-Menten constant (*K*_m_) and maximum initial velocity (*V*_max_) were calculated using the double reciprocal of the Michaelis-Menten equation, in which *K*m indicates the affinity of the nanozyme for the substrate (low *K*_m_ indicates a high affinity) and *V*_max_ represents the catalytic activity of the nanozyme [[54], [55], [56]]. The values of *K*_m_ and *V*_max_ were solved to be 21.1 mM and 0.73 × 10^−7^ M s^−1^, respectively, for the Zn SAs, whereas those of Janus Zn-Mo DAs were 9.6 mM and 1.2 × 10^−7^ M s^−1^, respectively. These results demonstrated that the Janus Zn-Mo DAs possessed higher reactivities and stronger affinities toward H_2_O_2_ than Zn SAs (Fig. 2a).

(2) A=εlc

(3) $$V_{0}=\frac{V_{\max}\cdot \left[S\right]}{K_{m}+\left[S\right]}$$

(4) $$\frac{1}{V_{0}}=\frac{K_{m}}{V_{\max}}.\frac{1}{\left[S\right]}+\frac{1}{V_{\max}}$$

In addition, the production of •OH was further confirmed by ESR measurements. As shown in Fig. S16, quartet intense characteristic ESR signals (1:2:2:1 in intensity) appeared using DMPO as the •OH-trapped agent, confirming •OH formation according to Equation (5), whereas a weaker ESR signal was observed for Zn SAs and no obvious signals were found for ZIF-8 alone. Fig. S17a illustrated the mechanism for TMB oxidation process. Moreover, the production of •OH increased with prolonging reaction time, indicating that Janus Zn-Mo DAs can effectively induce the decomposition of H_2_O_2_ to produce •OH due to the POD-like activity (Fig. S17b). The activation energies (*E*_a_) of Zn SAs and Janus Zn-Mo DAs were measured and computed using the Arrhenius equation to further explore the kinetic mechanism of H_2_O_2_ activation (Fig. S17c). The *E*_a_ was calculated to be 18.35 kJ mol^−1^ (Zn SAs) and 11.63 kJ mol^−1^ (Janus Zn-Mo DAs), which verified that asymmetric Zn-N_4_ and Mo-N_2_O_2_ moieties effectively reduced the energy barrier.

(5) Janus Zn-Mo DAs + H_2_O_2_ → •OH

Besides, negligible •O_2_^−^ signal was observed in the ESR spectrum for pure ZIF-8 (Fig. 2b). By contrast, the Zn SAs presented a weak peak with 1:1:1:1 ratio, demonstrating Zn SAs could transfer O_2_ to •O_2_^−^. Intriguingly, compared with Zn SAs, the Janus Zn-Mo DAs group exhibited much stronger •O_2_^−^ characteristic peak. Thus, the dual metal active site of Janus Zn-Mo DAs could produce abundant •O_2_^−^ according to the following Equation (6):

(6) Janus Zn-Mo DAs + O_2_ → •O_2_^−^

With the addition of 1,4-benzoquinone (BQ) to the Janus Zn-Mo DAs solution containing H_2_O_2_, the absorbance of methylene blue (MB) increased obviously due to the capture of •O_2_^−^ in the system, revealing that Janus Zn-Mo DAs can produce •O_2_^−^ (Fig. S18). In addition, the O_2_ generation experiment of Janus Zn-Mo DAs was conducted to confirm CAT activity. First, a titanium sulfate (Ti(SO_4_)_2_) reagent was employed to record changes in the H_2_O_2_ solution treated with Janus Zn-Mo DAs at different times (Fig. 2c). The O_2_ production experiments in the presence of H_2_O_2_ with or without Janus Zn-Mo DAs were displayed in Video S1 and Fig. S19. Compared to pure H_2_O_2_, obvious O_2_ bubbles were observed after adding Janus Zn-Mo DAs. Dissolved O_2_ concentration increased sharply from 0 to 6.78 mg L^−1^ in the acidic PBS containing GSH. Therefore, Janus Zn-Mo DAs were able to decompose H_2_O_2_ and efficiently convert H_2_O_2_ to O_2_ and H_2_O, thereby alleviating hypoxia in tumors. Notably, the production of O_2_ can also serve as the substrate for the OXD-like catalytic reaction, resulting in the fact that Janus Zn-Mo DAs can further convert O_2_ into •O_2_^−^. The charge transfer resistances of ZIF-8, Mo-ZIF8, Zn SAs and Janus Zn-Mo DAs were determined using electrochemical impedance spectroscopy (EIS). Compared to ZIF-8, Mo-ZIF8 and Zn SAs, Janus Zn-Mo DAs possessed the smallest impedance radius, revealing that the charge transfer resistance (R_ct_) was significantly reduced, which was attributed to the fast charge transfer between Zn and Mo atoms (Fig. 2d).

### 3.3 NIR-II activated photothermal properties

Benefiting from the pyrolysis of 2-methylimidazole, the UV–Vis–NIR absorption spectra of Janus Zn-Mo DAs were displayed in Fig. S20. Obviously, Janus Zn-Mo DAs dispersion solutions with various concentrations displayed stronger absorption at 1064 nm. Temperature changes of different concentrations (62.5, 125, 250, 500, and 1000 μg mL^−1^) for Janus Zn-Mo DAs gradually increased with the continuously increasing concentrations, indicating a significant concentration dependence between temperature change and concentration (Fig. 2e). A significant temperature elevation of 39.8 °C was observed in the Janus Zn-Mo DAs (1 mg mL^−1^) after exposing them to laser excitation (1.2 W cm^−2^) for 10 min. Furthermore, the temperature of the Janus Zn-Mo DAs solution showed a positive correlation with the laser power density and increased consistently as the power density increased (Fig. S21a). During the photothermal conversion process, stability and photothermal conversion efficiency are crucial factors. To further evaluate the photothermal stability of the Janus Zn-Mo DAs NPs, temperature changes in the Janus Zn-Mo DAs dispersion were monitored during five cycles of laser irradiation and natural cooling process. The results showed that the photothermal performance remained largely consistent after five heating and cooling cycles (Fig. S21b). The real-time curve corresponding to the heating and cooling processes for the Janus Zn-Mo DAs was shown in Fig. 2f. The corresponding photothermal conversion capabilities were estimated approximately 47.2 %. Subsequently, for the Janus Zn-Mo DAs, the negative natural logarithm of the time-temperature was obtained from the corresponding cooling curve. Notably, the absorbance of the colorimetric system was significantly increased when exposed to NIR light compared to dark conditions, demonstrating the photoenhanced enzymatic catalytic activity of the Janus Zn-Mo DAs (Fig. 2g).

Subsequently, to explore the influence of temperature on the catalytic performance of POD-like, CAT-like and OXD-like reactions, the nanozymes were placed at different temperatures (25 °C and 65 °C). First, to quantify the POD-like catalytic kinetics of the Janus Zn-Mo DAs at different temperatures, a steady-state kinetic assay was performed (Fig. S22). As presented in Fig. 2h and Table S3, the *K*_m_ values for H_2_O_2_ as substrates for Janus Zn-Mo DAs (65 °C) were lower than those of Janus Zn-Mo DAs (25 °C) and Zn SAs (25 °C), indicating that Janus Zn-Mo DAs (65 °C) exhibited better binding affinity to substrates due to the temperature increased. Meanwhile, catalytic efficiency (*k*_cat_/*K*_m_) values calculated based on the Fe active sites for these nanozymes revealed a notable 1.4 and 5.1-fold increase for Janus Zn-Mo DAs (65 °C) compared to Janus Zn-Mo DAs (25 °C) and Zn SAs (25 °C), respectively, showing that the hyperthermia could augment POD-like activities. Then, the O_2_ generation was monitored at 25 °C and 65 °C to explore the effect of temperature on CAT-like enzyme activity. The O_2_ production rate was significantly enhanced at 65 °C (Video S2), which further confirmed that the higher the temperature, the stronger the CAT enzyme activity. In addition, the effect of temperature on OXD-like activities was shown in Fig. 2i. With the addition of BQ to the Janus Zn-Mo DAs (25 °C) and Janus Zn-Mo DAs (65 °C) solution containing H_2_O_2_, the absorbance of methylene blue (MB) for Janus Zn-Mo DAs (65 °C) changed significantly compared with that of Janus Zn-Mo DAs (25 °C), revealing that Janus Zn-Mo DAs (65 °C) could produce more •O_2_^−^. As shown in Fig. S23, the yield of •O_2_^−^ was nearly identical under both laser irradiation and conventional heating. The result demonstrates that the generation of •O_2_^−^ is primarily enhanced by the photothermal effect, rather than by laser-induced electron-hole pair formation. Therefore, all these enzyme-mimicking catalytic activities of Janus Zn-Mo DAs could be boosted evidently under hyperthermia (65 °C).

### 3.4 DFT calculations and enzymatic catalysis mechanism

**Fig. 3:**
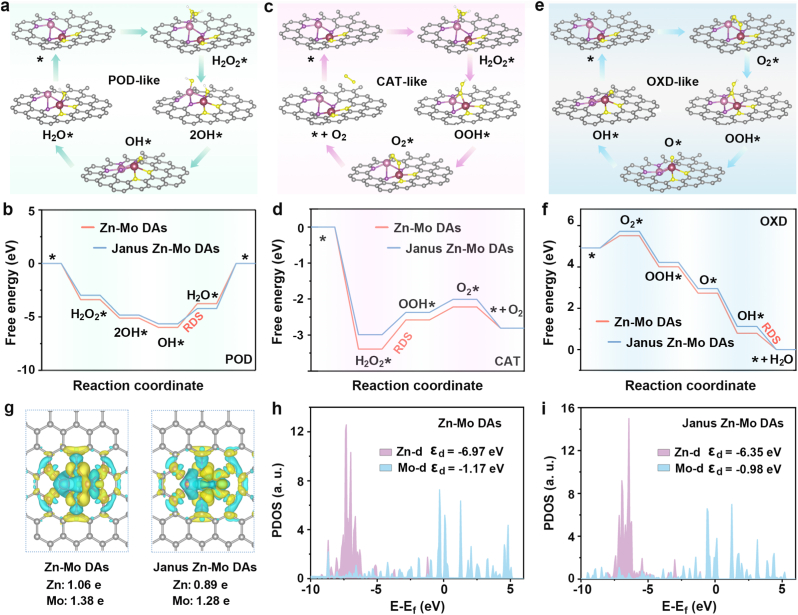
Catalytic mechanism (a) and free-energy diagrams (b) of the POD-like reaction path on Janus Zn-Mo DAs. Catalytic mechanism (c) and free-energy diagrams (d) of the CAT-like reaction path on Janus Zn-Mo DAs. Catalytic mechanism (e) and free-energy diagrams (f) of the OXD-like reaction path on Janus Zn-Mo DAs. Charge density differences and Bader charges of Zn-Mo DAs and Janus Zn-Mo DAs (g). PDOS of Zn-Mo DAs (h) and Janus Zn-Mo DAs models (i).

DFT calculations were performed to obtain a comprehensive understanding of the catalytic mechanisms of POD, CAT, and OXD-like catalytic reactions. According to the XAFS and XPS results, the local atomic configurations of the active centers of N_4_-Zn-Mo-N_4_ and N_4_-Zn-Mo-N_2_O_2_ corresponding to the Zn-Mo DAs and Janus Zn-Mo DAs, respectively, were constructed and optimized (Fig. S24). As shown in Fig. 3a and S25a, the energy profiles of POD-like catalytic properties were determined, where H_2_O_2_ molecules were transformed into hydroxyl radicals. The initial step of the reaction was the H_2_O_2_ adsorption on the catalyst carrier. The dissociation of H_2_O_2_ on Zn-Mo DAs and Janus Zn-Mo DAs was highly exothermic with free energies by 3.39 and 2.99 eV, respectively. Next, the activated H_2_O_2_∗ molecules were uniformly cleaved into two hydroxyl groups (OH∗), followed by OH∗ desorption at the catalytic site to form •OH radicals. Under acidic conditions, the adsorbed OH∗ intermediate interacted with H^+^ to form H_2_O∗. This process was slightly uphill in the free-energy profile, which is a rate-determining step (RDS) [57]. Comparatively, the Janus Zn-Mo DAs exhibited good catalytic activity than that of the Zn-Mo DAs because of its lower energy barrier (1.43 eV vs. 2.21 eV) (Fig. 3b). After the desorption of H_2_O molecules, the nanozymes returned to its initial states. The recovery of the nanozymes continued to activate another H_2_O_2_ molecules to generate •OH in subsequent cycles.

Similarly, for the CAT-like catalytic activity of Janus Zn-Mo DAs, we optimized the reaction pathway and calculated the free energy change along the optimal reaction path for each of the two nanozymes (Fig. 3c and Fig. S25b). For the pathways of CAT-like reaction, the adsorption of H_2_O_2_ molecules at the Zn sites was the initial step. In the second step, H_2_O_2_∗ dissociated into OOH∗ species. Subsequently, the OOH∗ species underwent H transformation to generate O_2_. As shown in Fig. 3d, the step of OOH∗ formation was the RDS of the entire O_2_ production process. The energy barrier was calculated to be 0.81 and 0.62 eV, respectively. The lowest energy barrier of the Janus Zn-Mo DAs indicated that they can efficiently catalyze the generation of O_2_. For the OXD-like reaction (Fig. 3e and Fig. S25c), the first step is O_2_ adsorption and then hydrogenation to generate OOH∗ species. This species was further hydrogenated and dehydrated to form the O∗ species, and subsequently underwent two consecutive hydrogenation steps to obtain an H_2_O molecule. The RDS in OXD-like reaction is the desorption process of adsorbed OH∗ intermediate. The overpotential was respectively calculated to be 0.44 and 0.11 eV for Zn-Mo DAs and Janus Zn-Mo DAs, respectively, indicating that Janus Zn-Mo DAs are easier to improve the catalytic efficiency of OXD-like reaction than Zn-Mo DAs (Fig. 3f). The schematic illustrations of the distinct catalytic mechanisms (POD, CAT, OXD-like) were displayed in Fig. S25d. These findings indicated that the robust synergistic interactions between the Zn-N_4_ and Mo-N_2_O_2_ moieties could effectively reduce the energy barriers for POD-like, CAT-like, and OXD-like reactions by modulating the charge distribution of active sites, thus resulting in significantly improved catalytic activities for these reactions.

As shown in Fig. 3g, the charge density differences of the optimal models of Zn-Mo DAs and Janus Zn-Mo DAs *via* first-principle calculations were constructed and calculated for comparison, where sky-blue and yellow parts represented the depletion and accumulation of electrons, respectively. Differential charge density analysis revealed the accumulation of negative charges near the Zn and Mo atoms in the Janus Zn-Mo DAs, which implied the redistribution of electrons in the Janus Zn-Mo DAs. In addition, the results of Bader charge demonstrated that the charge of Zn/Mo atoms in Janus Zn-Mo DAs (0.89 e/1.28 e) was slightly lower than that in the Zn-Mo DAs (1.06 e/1.38 e), suggesting a relatively weaker electron depletion. According to previous studies, the increased electron localization around the metal sites in asymmetric configurations can decrease the energy barriers associated with the formation of intermediate [58]. The partial density of states (PDOS) for the Zn-Mo DAs and Janus Zn-Mo DAs were calculated, and the results showed that the Zn and Mo atoms in the Janus Zn-Mo DAs exhibited a higher charge density near the Fermi level compared to the Zn-Mo DAs, thereby facilitating electron transport in the reaction process (Fig. 3h and i). Importantly, the d-band center of Zn 3d up-spin orbital (−6.35 eV) and Mo 3d up-spin orbital (−0.98 eV) of Janus Zn-Mo DAs were much closer to the Fermi level than those of Zn-Mo DAs, respectively. The results revealed that Janus Zn-Mo DAs could induce electron redistribution at the active center, thereby breaking the charge localization of the Zn 3d and Mo 3d orbitals, decreasing the rate-limiting step energy barriers for the reaction process and further improving the catalytic activity.

### 3.5 *In vitro* evaluation of biocompatibility and anti-cancer efficacy

**Fig. 4:**
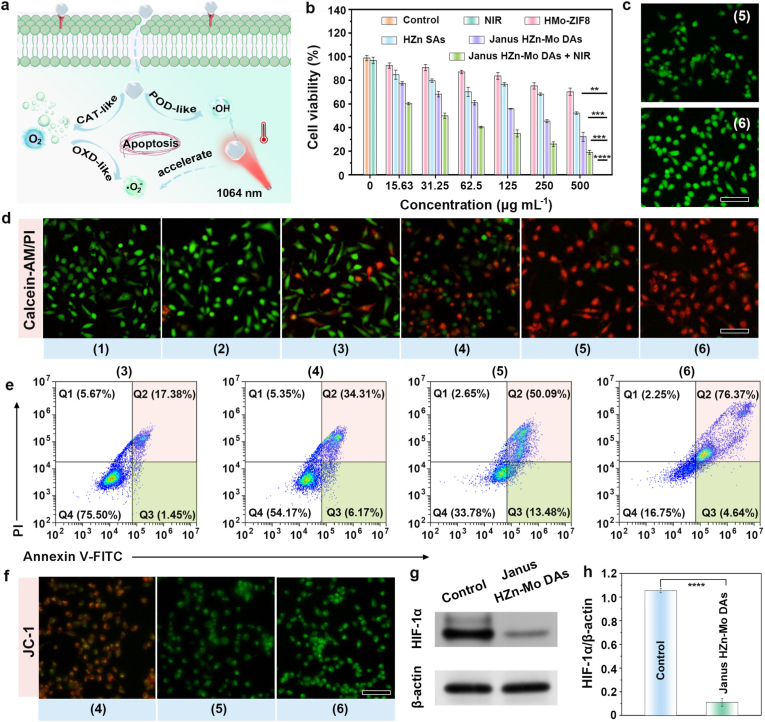
Schematic of apoptosis induced in cells by the Janus HZn-Mo DAs treatment (a). Cytotoxicity assessment of HeLa cells after various treatments by MTT assay. Statistical analysis was performed *via* one-way ANOVA with a Bonferroni multiple comparisons post hoc test. Error bars denote the standard deviation (n = 3, mean ± SD, ∗∗*p* < 0.01, ∗∗∗*p* < 0.001, ∗∗∗∗*p* < 0.0001) (b). Intracellular ROS measurement by CLSM images of DCFH-DA staining after treatments with (5) Janus HZn-Mo DAs, or (6) Janus HZn-Mo DAs plus NIR (1064 nm, 1 W cm^−2^) for 10 min. Scale bar: 50 μm (c). CLSM images of HeLa cells stained with calcein-AM (green)/PI (red) after different treatments: (1) PBS (Control), (2) PBS plus 1064 nm (NIR), (3) HMo-ZIF8, (4) HZn SAs, (5) Janus HZn-Mo DAs, or (6) Janus HZn-Mo DAs plus NIR. Scale bar: 50 μm (d). Flow cytometry in HeLa cells after different treatments (e). JC-1 staining of HeLa cells receiving various treatments. Scale bar: 50 μm (f). Western blot of HIF-1α after varied treatments (g). Quantitative ratio of HIF-1α to β-actin in each group. Statistical significance is assessed by unpaired Student's two-sided *t*-test, ∗∗∗∗*p* < 0.0001. Data were defined as mean ± S.D. (n = 3) (h).

Additionally, hyaluronic acid (HA) was chosen to enhance the biocompatibility of the Janus Zn-Mo DAs. As shown in Fig. S26, the peaks at 1624 cm^−1^ corresponded to the stretching vibration of -C=O- stretching vibration, implying successful modification of HA. Furthermore, the observed charge reversal in zeta potential confirmed the successful modification of HA (Fig. S27). Considering the good catalytic effects of the Janus HZn-Mo DAs, the cell apoptosis mechanism was investigated (Fig. 4a). These results suggested that Janus HZn-Mo DAs can generate a significant amount of reactive oxygen species (ROS) in cancer cells through multiple enzyme-catalyzed reactions. Furthermore, as depicted in Fig. S28, hemolysis tests showed no obvious hemolysis of red blood cells (RBCs) at the tested dose, further confirming the good biocompatibility of Janus HZn-Mo DAs. The viability of Janus HZn-Mo DAs at the cellular scale was evaluated by the 4,5-dimethylthiazol2-yl-2,5-diphenyl tetrazolium bromide (MTT) assay. As the concentration of Janus HZn-Mo DAs varied from 0 to 500 μg mL^−1^, the survival rate of L929 cells remained above 94 % in each group, indicating that Janus HZn-Mo DAs exhibited negligible cytotoxicity (Fig. S29a). The biosafety of Janus HZn-Mo DAs was evaluated using a live/dead cell co-staining assay with calcein-acetoxymethyl ester (Calcein-AM) and propidium iodide (PI). The results showed that almost all regions exhibited green fluorescence, indicating that no distinctive damage was detected in the L929 cells (Fig. S29b). Confocal laser scanning microscope (CLSM) images were employed to elucidate the phagocytosis of the HeLa cells treated with FITC-modified Janus HZn-Mo DAs. Fig. S29c and Fig. S30 showed the recorded green and blue fluorescence signals in the cytoplasm and nuclei of HeLa cells, respectively, suggesting that Janus HZn-Mo DAs were efficiently internalized by HeLa cells compared with normal cells. In addition, bio-TEM was used to confirm the cellular phagocytosis of HeLa cells treated with Janus HZn-Mo DAs for 1 h (Fig. S31). A substantial quantity of Janus HZn-Mo DAs was observed in HeLa cells, which was essential for the catalytic performance of nanozymes in targeting and eliminating cancer cells.

The viability of HeLa cells was evaluated using Janus HZn-Mo DAs-mediated catalytic therapy (Fig. 4b). The survival rate of HeLa cells in the 1064 group was 97 %, revealing no obvious damage to HeLa cells after 1064 nm-laser irradiation. Similarly, HeLa cells maintained a high survival rate in the HMo-ZIF8 and HZn SAs groups. However, as the concentration of Janus Zn-Mo DAs increased from 0 to 500 μg mL^−1^, the production of •OH was elevated. This increase led to a gradual decrease in the survival rate of HeLa cells. Compared to the Janus HZn-Mo DAs alone (500 μg mL^−1^), the HeLa cells survival rate incubated with Janus HZn-Mo DAs plus 1064 nm laser irradiation (1.0 W cm^−2^) decreased from 32.2 % to 19.1 %, respectively. All data suggested that Janus HZn-Mo DAs exhibited the strongest killing effect on HeLa cells when combined with 1064 nm laser irradiation, which was attributable to the synergistic effect of hyperthermia-augmented nanocatalytic therapy.

Generally, cell death is closely associated with intracellular ROS production [59]. The 2,7-dichlorofluorescein diacetate (DCFH-DA) was used as ROS probe to assess ROS formation [60,61]. No obvious green fluorescence traces were observed in both the control and 1064 nm groups. As shown in Fig. S32a and Fig. 4c, the HeLa cells treated with HMo-ZIF8 and HZn SAs exhibited weak fluorescence intensity. However, the Janus HZn-Mo DAs group demonstrated enhanced green fluorescence owing to the production of ROS induced by the highly active Zn and Mo centers, conforming to the free radical capture experiment. Conversely, the Janus HZn-Mo DAs plus 1064 nm group presented the strongest fluorescence intensity owing to the hyperthermia-augmented catalytic therapy. As shown in Fig. S32b, the fluorescence intensity in the Janus Zn-Mo DAs plus 1064 nm laser irradiation group was 2.0-fold higher than that of the Janus Zn-Mo DAs group, indicating that the NIR-II laser stimulation promoted the catalytic ROS production. Subsequently, the cancer cell-killing efficiency of Janus HZn-Mo DAs was evaluated using a live/dead cell co-staining assay with alcein-acetoxymethyl ester (Calcein-AM) and propidium iodide (PI) following various treatments [62]. In the control and 1064 nm laser irradiation groups, almost all regions exhibited green fluorescence, indicating that no distinctive damage was detected in the HeLa cells (Fig. 4d). Simultaneously, the HeLa cells incubated with HMo-ZIF8 and HZn SAs showed weak green fluorescence. In contrast, Janus HZn-Mo DAs presented numerous red regions because of the dual active-sites-mediated catalytic therapy. In comparison with other groups, the highest red fluorescence revealed that Janus HZn-Mo DAs plus 1064 nm had the best cytotoxic effect, which was attributable to hyperthermia-augmented catalytic therapy.

In addition, we performed a control experiment using ROS scavenger N-acetyl-L-cysteine (NAC) to analyze the anticancer contribution of photothermal and catalytic effects. As shown in Fig. S33, when ROS are scavenged, the therapeutic potency is almost abolished. Namely, the catalytic generation of ROS is the dominant mechanism. Furthermore, the viability of HeLa cells in the different treatment groups was investigated using a flow cytometric apoptosis assay (Fig. S34 and Fig. 4e). The overall percentages of necrosis in the control, 1064 nm, HMo-ZIF8, HZn SAs, Janus HZn-Mo DAs and Janus HZn-Mo DAs plus 1064 nm groups were 3.98, 5.84, 18.83, 40.48, 63.57 and 81.01 %, respectively. The Janus HZn-Mo DAs plus 1064 nm group exhibited the strongest inhibition rate of HeLa cells compared with that in the other groups, in accordance with the MTT results. Previous studies have shown that cellular damage is closely related to mitochondrial dysfunction. Therefore, the killing mechanism of Janus HZn-Mo DAs-induced mitochondrial membrane potential damage was further studied. JC-1 staining was performed to assess its effects on mitochondrial function. Notably, JC-1 primarily showed red aggregate fluorescence in normal mitochondria with high membrane potential, whereas it showed green monomer fluorescence in damaged mitochondria with low membrane potential [63]. As illustrated in Fig. S35 and Fig. 4f, the strongest red aggregate signal appeared in HeLa cells after treatment with the control and 1064 nm groups, suggesting that the mitochondrial membrane potential was basically intact. In particular, compared to the Janus HZn-Mo DAs group, HeLa cells incubated with Janus HZn-Mo DAs plus 1064 nm irradiation demonstrated the strongest green monomer fluorescence, indicating that the mitochondrial membrane potential of the NIR-II-irradiated Janus HZn-Mo DAs therapeutic agents was largely depolarized. Moreover, hypoxia in the tumor microenvironment subjected to different treatments was estimated by staining the extracted tumor tissues with hypoxia inducible factor-1α (HIF-1α). As shown in Fig. 4g and h, the Janus HZn-Mo DAs groups exhibited a reduced HIF-1α expression, thereby indicating that the Janus HZn-Mo DAs may produce O_2_ and alleviate hypoxia.

### 3.6 *In vivo* catalytic therapy

**Fig. 5:**
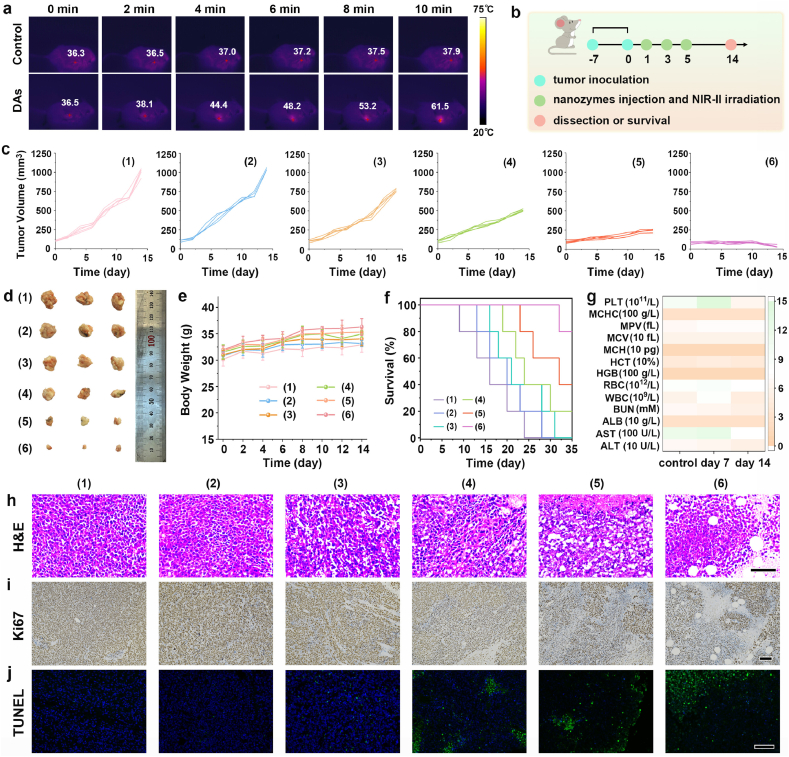
The thermal images of U14 tumor-bearing mice in different groups after 10 min NIR-II laser irradiation (a). Scheme for the schedule of *in vivo* Janus HZn-Mo DAs treatment (b). Tumor volume growth curves (c), photographs of representative excised tumor (d), body weight (e) and Kaplan-Meier survival curves (f) of tumor-bearing mice after different treatments: (1) PBS (Control), (2) PBS plus 1064 nm (NIR), (3) HMo-ZIF8, (4) HZn SAs, (5) Janus HZn-Mo DAs, and (6) Janus HZn-Mo DAs plus NIR. Data were presented as mean ± S.D. (n = 5). Hematological indexes and biochemical data of mice after intravenous injection with PBS and Janus HZn-Mo DAs for 7, and 14 days (g). H&E (h), Ki67 (i) and TUNEL (j) staining of tumor sections from the tumor-bearing mice in different treatment groups. Scale bar: 100 μm.

Fig. 5a illustrated that the temperature in the tumor region treated with Janus HZn-Mo DAs increased by approximately 25 °C within 10 min after 1064 nm laser irradiation. Conversely, the temperature in the tumor region exposed to only 1064 nm laser irradiation increased minimally under the same conditions. These findings verified that Janus HZn-Mo DAs possessed a substantial photothermal effect. Furthermore, the change of tumor temperature after intravenous injection of nanozymes during treatment was explored. For Janus HZn-Mo DAs group, the tumor temperature of mice reached 41.4 °C after 10 min, while the NIR irradiation group was only 36.1 °C at the same time point (Fig. S36). The difference in temperature between the two injection methods is due to the difference in the amount of sample retained at the tumor site. Therefore, the photothermal effect is insufficient for antitumor in our system, and the tumor suppression effect is derived from heating-enhanced catalytic effect. The corresponding therapeutic efficacy of Janus HZn-Mo DAs in inhibiting tumor growth was systematically evaluated using U14 tumor-bearing mice, which benefited from the high killing ability of cancer cells (Fig. 5b). First, the U14 animal models were randomly separated into six groups, including control, 1064 nm laser irradiation, HMo-ZIF8, HZn SAs, Janus HZn-Mo DAs and Janus HZn-Mo DAs plus 1064 nm to investigate the antitumor performance. The mice treated with PBS or 1064 nm laser groups presented rapid tumor growth (Fig. 5c). By contratst, an obvious tumor-suppression effect was observed in the Janus HZn-Mo DAs group, indicating a favorable enzyme-catalyzed therapeutic effect is due to the high performance of multiple enzymes. Compared with other groups, Janus HZn-Mo DAs therapeutic agents combined with 1064 nm laser irradiation displayed the highest killing effect on tumors because of photothermal enhanced catalytic activities. The photothermal effect not only increased the local temperature of the tumor region, but also improved the efficacy of optothermal-involved catalytic process.

As shown in Fig. 5d, digital pictures of the dissected tumors were obtained after the therapeutic period. Compared with the control and 1064 nm groups, there is almost no tumor suppression effect was observed in the HMo-ZIF8, and HZn SAs groups. In contrast, Janus HZn-Mo DAs exhibited tumor suppression effect clearly, owing to the dual active sites-mediated catalytic therapy. However, when Janus HZn-Mo DAs were illuminated at 1064 nm, Janus HZn-Mo DAs plus 1064 nm showed the highest antitumor effect because of the hyperthermia-augmented tumor catalytic therapy. Throughout the treatment period, no significant differences in body weight changes were observed among all the mice, indicating that the treatment modality was highly biocompatible (Fig. 5e). In addition, the Janus HZnMo-DAs plus 1064 nm irradiation group showed a significantly extended survival time of tumor-bearing mice compared to the other treatment groups after 35 days (Fig. 5f). During this period, there were no abnormal changes or fluctuations in the activity and dietary status, living habits as well as the body weights of the mice for Janus HZn-Mo DAs plus NIR group. Additionally, the good biological safety of the Janus HZn-Mo DAs was confirmed by blood routine and biochemical analyses (Fig. 5g). The tumor accumulation of the Janus HZn-Mo DAs was higher than that of Janus Zn-Mo DA, indicating the better targeting nature of HA-modified nanozyme (Fig. S37). The dominant clearance route is speculated to be *via* the hepatobiliary system, which is consistent with the biodistribution data.

Hematoxylin and eosin (H&E) staining of tumor sections from each group were performed to reveal cell necrosis. Compared with that in the other groups, typical histopathological damage was easily observed in the HZn SAs, Janus HZn-Mo DAs and Janus HZn-Mo DAs plus 1064 nm groups (Fig. 5h). Subsequently, no significant organ damage or pathological abnormalities were evident in the major organs (heart, liver, spleen, lung, and kidney), suggesting the good biocompatibility of Janus HZn-Mo DAs therapeutic agents (Fig. S38). Additionally, immunohistochemistry staining analysis was further employed to understand the antitumor mechanism. Tumor apoptosis and proliferation were assessed using Ki67 staining and terminal deoxynucleotidyl transferase-mediated deoxyuridine triphosphate nick end labeling (TUNEL) immunofluorescence staining (Fig. 5i and j). In the Janus HZn-Mo DAs plus 1064 nm group, the tumor sections stained with Ki67 and TUNEL displayed that a large number of tumor cells had the lowest level of cell proliferation and the strongest cell apoptotic signaling compared with other groups. These results confirmed that Janus HZn-Mo DAs plus 1064 nm showed the highest inhibitory effects on tumor cells through the synergistic effect of dual active sites-mediated catalytic therapy and optothermal-involved catalytic therapy. The above results also revealed that Janus HZn-Mo DAs could serve as effective therapeutic agents owing to the production of multiple ROS in the tumor microenvironment.

## 4 Conclusions

In summary, a simple strategy for synthesizing Janus Zn-Mo dual-atomic sites was developed to optimize the adsorption/desorption of O-containing reaction intermediates during POD, CAT and OXD-like enzyme processes, thereby improving the efficacy of tumor catalytic therapy. Benefiting from the distinctive configurations and synergistic effects of the Zn-N_4_ and Mo-N_2_O_2_ moieties, the as-synthesized Zn-Mo DAs exhibited exclusive multi-enzyme catalytic activities. The relationship between multi-enzyme performance and the coordination environments of nanozymes was precisely elucidated by the combination of experimental investigation and DFT calculations. Compared with non-Janus structured DAs, the enzymatic catalytic activities of Janus DAs were improved, which was attributed to the fact that the Janus structure regulated the d-band center of active sites and tuned the reaction intermediates by the enzymatic catalytic process. Simultaneously, the obtained Zn-Mo DAs exhibited the photothermal conversion efficiency of 47.2 % when exposed to 1064 nm laser. The antitumor experiments validated that Janus HZn-Mo DAs can efficaciously induce tumor cell apoptosis, achieving tumor inhibition rate up to 95.0 % with ignorable damage to normal tissues. Together, this study provides a simple and efficient strategy for designing dual-atom active sites on carbon substrates for high-performance nanozymes.

## CRediT authorship contribution statement

**Shuang Liu:** Writing – original draft, Data curation, Conceptualization. **Jiawei Qu:** Formal analysis, Data curation. **Jiating Xu:** Writing – review & editing, Funding acquisition, Conceptualization. **Qiang Wang:** Data curation. **Le Zhang:** Investigation. **Xinyu Zhang:** Methodology. **Chunsheng Li:** Investigation. **Yong Lu:** Data curation. **Yi Zhong:** Resources. **Linbo Li:** Resources, Formal analysis. **Piaoping Yang:** Writing – review & editing, Resources, Formal analysis.

## Declaration of competing interest

The authors declare that they have no known competing financial interests or personal relationships that could have appeared to influence the work reported in this paper.

## Data Availability

Data will be made available on request.

## Appendix A Supplementary data

The following arec the supplementary data to this article:
