# Supplementary material for: Janus Zn-Mo nanozymes for leveraged trienzymatic anticancer therapy
Source: Mater Today Bio. 2025 Dec 9;36:102662. doi: 10.1016/j.mtbio.2025.102662 (PMC12765334; doi:10.1016/j.mtbio.2025.102662)
Supplement: Multimedia component 1 [file mmc1.docx]

Janus Zn-Mo nanozymes for leveraged trienzymatic anticancer therapy

Shuang Liu ^a,b^, Jiawei Qu ^a^, Jiating Xu ^a,b,^*, Qiang Wang ^a^, Le Zhang ^a^, Xinyu Zhang ^a^, Chunsheng Li ^a^, Yong Lu ^a^, Yi Zhong ^c^, Linbo Li ^c,^*, and Piaoping Yang ^d,^*

^a^ *Key Laboratory of Forest Plant Ecology, Ministry of Education, College of Chemistry, Chemical Engineering and Resource Utilization, Northeast Forestry University, Harbin 150040, P. R. China*

^b^ *Heilongjiang Provincial Key Laboratory of Ecological Utilization of Forestry-Based Active Substances, Northeast Forestry University, Harbin, 150040, P. R. China*

^c^ *Faculty of Materials Science and Energy Engineering/Institute of Technology for Carbon Neutrality, Shenzhen Institute of Advanced Technology, Chinese Academy of Sciences, Shenzhen, 518055, P.R. China*

^d^ *Key Laboratory of Superlight Materials and Surface Technology, Ministry of Education, College of Material Sciences and Chemical Engineering, Harbin Engineering University, Harbin, 150001, P. R. China*

*Corresponding author.

E-mail addresses: xujiating@nefu.edu.cn (J. Xu), lb.li@siat.ac.cn (L. Li), yangpiaoping@hrbeu.edu.cn (P. Yang)

1. Materials and methods

*1.1. Chemicals and reagents*

Zinc nitrate hexahydrate (Zn(NO_3_)_2_·6H_2_O) and 2-Methylimidazole (MeIM) were obtained from the Tianjin Fengchuan Chemical Reagent Co., Ltd., Molybdenyl (VI) oxide bis(2, 4pentanedionate) (Mo(acac)_3_) was gained from Shanghai DiBo Biochemical Co., Ltd. Hyaluronic acid (HA), methylene blue (MB), 3,3',5,5'-tetramethylbenzidine (TMB), 5,5-dimethyl-1pyrroline N-oxide (DMPO), 2,2,6,6-tetramethyl-4-piperidone (TEMP), titanium oxide sulfate (TiOSO_4_), 3-(4,5-dimethyl-2-thiazolyl)-2,5-diphenyl-2-H-tetrazolium bromide (MTT), fluorescein isothiocyanate (FITC), calcein AM and propidium iodide (PI) and 4′,6-diamidino-2-phenylindole (DAPI) were obtained from the Aladdin Reagents Company Co., Ltd. 1,4-benzoquinone (BQ) were purchased from RHAWN Chemical Agents. Phosphate buffer solution (PBS) (pH 7.4), phosphate buffer solution (PBS) (pH 6.5) and fetal bovinef serum (FBS) were purchased from Beyotime Biotechnology Co., Ltd., and methanol (MeOH), ethanol (C_2_H_5_OH) and dimethyl sulfoxide (DMSO) were got from Tianjin Institute of Chemical Agents. Dulbecco's Modified Eagle Medium (DMEM) was received from HyClone. 1,3-Diphenylisobenzofuran (DPBF), 2′,7′-dichlorodihydrofluorescein diacetate (DCFH-DA), hydrogen peroxide (H_2_O_2_, 30%), Mitochondrial Membrane Potential Kit (JC-1 Assay) and Annexin V-FITC Apoptosis Detection Kit were ordered from Beijing Solarbio Science & Technology Co., Ltd.. Distilled water was used in the experiment. All of the chemical reagents were used without further purification.

*1.2.* *Characterization*

The morphology of the sample was analyzed by scanning electron microscope (SEM, JSM-7500F) and transmission electron microscope (JEM-2100) (Japan electronics). X-ray powder diffractometer (Bruker D8 ADVANCE) was employed to investigate the crystallographic properties of the catalyst. Data analysis was supported by Zhongke e-Test Research Service (www.zkec.cc). The high-angle annular dark-field scanning transmission electron microscope (HAADF-STEM, Thermo Scientific, Themis Z) was employed to observe the existence of single atom. Selected area electron diffraction (SAED) patterns were obtained with a JEM-2100 transmission electron microscope. Energy disperse X-ray (EDX) was performed on a field emission scanning electron microscope (JSM-7500F) to analyze elemental features of the samples. The chemical composition and element valence were analyzed using X-ray photoelectron spectroscopy (XPS, Kratos Axis Ultra) with a monochromated Al Kα X-ray source (*h*ν = 1486.6 eV). N_2_ adsorption-desorption isotherm measurements were investigated to study the Brunauer-Emmett-Teller (BET) specific surface areas of the samples. Zn *K*-*edge* and Mo *K*-edge EXAFS and XANES spectra were performed with Si (111) crystal monochromators at the BL11B beamlines at the Shanghai Synchrotron Radiation Facility (SSRF) (Shanghai, China). The ESR spectra were examined on a Bruker ELEXSYS-II E500 CW-EPR. The infrared spectra were measured using a Fourier-transform infrared spectrometer spectrum 400 (PerkinElmer) within the range of wavenumbers 4000-550 cm^-1^. The ultraviolet-visible-infrared (UV-Vis-NIR) spectrometry was measured on UH5700 spectrophotometer. Electrochemical characterizations were performed on an electrochemical workstation (CHI660E) using a three-electrode system (pH 7.0) with Pt as the counter electrode, Ag/AgCl as the reference electrode under visible light. The electrolyte was 1 M Na_2_SO_4_ aqueous solution. Elemental analyses were conducted on inductively coupled plasma-optical emission spectrometry (ICP-OES) (Agilent 5110). The cell fluorescence imaging experiments were carried out using confocal laser scanning fluorescence microscope (CLSM). Cell viabilities were measured by recording the absorption at 490 nm by using a Microplate Reader. The flow cytometry data was obtained by CytoFLEX S. Dimensions dispersion were obtained by a dynamic light scattering (DLS) instrument (Zetasizer Nano ZS, Malvern Panalytical, UK).

*1.3. Computational methods*

We have employed the Vienna Ab Initio Package (VASP) [1,2] to perform all the density functional theory (DFT) calculations within the generalized gradient approximation (GGA) using the PBE formulation [3,4]. We have chosen the projected augmented wave (PAW) potentials to describe the ionic cores and take valence electrons into account using a plane wave basis set with a kinetic energy cutoff of 450 eV. Partial occupancies of the Kohn-Sham orbitals were allowed using the Gaussian smearing method and a width of 0.05 eV. The electronic energy was considered self-consistent when the energy change was smaller than 10^-4^ eV. A geometry optimization was considered convergent when the force change was smaller than 0.05 eV Å^-1^. Grimme’s DFT-D_3_ methodology was used to describe the dispersion interactions. The Brillouin zone integral uses the surfaces structures of 2 × 2 × 1 monkhorst pack K-point sampling. Finally, the adsorption energies (*E_ads_*) were calculated as:

*E_ads_* = *E_ad_/_sub_* -*E_ad_ - E_sub_* (1)

where *E_ad_/_sub_*, *E_ad_*, and *E_sub_* are the total energies of the optimized adsorbate/substrate system, the adsorbate in the gas phase, and the clean substrate, respectively. The free energy (*ΔG*) for elemental reaction steps were calculated as:

*ΔG* = *ΔE* + *ΔE_ZPE_*- *TΔS* (2)

where *ΔE* is the difference between the total energy, *ΔE_ZPE_* and *ΔS* are the differences in the zero-point energy and the change of entropy, *T* is the temperature (*T* = 300 K in this work), respectively. The *U* correction had been adopted in our systems, and fixed the section atoms.

*1.4. Preparation of ZIF-8*

A typical synthesis of ZIF-8 was performed as follows: 2-methylimidazole (3.7 g) was dissolved in 80 mL methanol with sonification for 30 min to form a homogeneous solution in flask A; Zn(NO_3_)_2_·6H_2_O (1.67 g) was dissolved in 40 mL methanol under ultrasound for 15 min to form a clear solution in flask B. Then, the mixture in flask B was dropwise added into flask A with vigorous stirring for 12 h at room temperature. The obtained product was separated by centrifugation and washed subsequently with methanol for three times and finally dried overnight at 60 °C under vacuum.

*1.5. Preparation of Zn SAs*

The Zn SAs can be prepared by thermal treating the as-prepared ZIF-8 sample. In a typical procedure, the prepared ZIF-8 precursor was vividly placed in a tube furnace and heated to 800 °C for 3 h with a heating rate of 5 °C min^-1^ under flow nitrogen gas. Then, the sample was naturally cooled down to the room temperature.

*1.6. Measurement of the peroxidase (POD)-like activity of Janus Zn-Mo DAs*

POD-like performance assays of Janus Zn-Mo DAs (200 μg mL^–1^) were conducted using TMB (400 μM) as substrates in the presence of H_2_O_2_ (400 mM) in PBS solution (pH 6.5). After a certain reaction time, the absorbance of the color reaction was collected by UV-vis spectra. Additionally, in order to further verify that photothermal effects can promote the generation of •OH, TMB chromogenic reactions at different temperatures (25 and 65 °C) were performed.

The steady-state kinetic assays were performed in 3 mL PBS solution (pH 6.5) with Zn SAs or Zn-Mo DAs (100 μg mL^-1^) as a catalyst in the presence of H_2_O_2_ and TMB. Meanwhile, the kinetic assays of Zn SAs or Zn-Mo DAs using H_2_O_2_ as substrate (5, 10, 20, and 40 mM) were also performed. All reactions were monitored by measuring the absorbance after different reaction times, and the Michaelis-Menten constant was calculated based on the Michaelis-Menten saturation curve. The following Equation was used to calculate the nanozyme activity:

A=*εlc* (3)

$v_{0}$ = $\frac{V_{max}\cdot[S]}{K_{M} + [S]}$ (4)

$\frac{1}{v_{0}}$ = $\frac{K_{m}}{V_{max}}\cdot\frac{1}{[S]}+\frac{1}{V_{max}}$ (5)

where *v_0_* is the initial velocity, *ε* is the molar absorption coefficient of the colorimetric substrate, *l* is the path length of the cuvette (cm), *[S]* is the concentration of the substrate, *K_m_* is the Michaelis-Menten constant, and *V*_max_ is the maximal reaction velocity.

*1.7. Measurement of the catalase (CAT)-like activity of Janus Zn-Mo DAs*

CAT-like activities of Zn-Mo DAs were evaluated at 25 °C and 65 °C by detecting the O_2_ production. Specifically, 1 mL H_2_O_2_ was mixed with 2 mL PBS (pH 6.5), followed by the addition of 1 mL Janus Zn-Mo DAs (1.0 mg mL^–1^) at 25 °C and 65 °C, respectively. As a control, the other group was only added with H_2_O_2_. Then, the experimental phenomenon of oxygen bubbles was recorded by taking videos.

In addition, the CAT-like activity was evaluated by measuring the O_2_ production. Specifically, 1 mL H_2_O_2_ (30 mM) was mixed with 8 mL PBS (pH 6.5) and PBS (pH 6.5) with 10 mM GSH, followed by the addition of 1 mL nanozymes (Janus Zn-Mo DAs, 200 μg mL^-1^).

*1.8. Measurement of the oxidase (OXD)-like activity of Janus Zn-Mo DAs*

Briefly, Janus Zn-Mo DAs (7 mg) and MB (7 mg) were added to 7 mL of deionized water to obtain a mixture solution. Subsequently, 1 mmol of BQ was added to the above mixture. Then, the mixed solution was divided into six groups: Janus Zn-Mo DAs (25 °C), Janus Zn-Mo DAs (65 °C) and Janus Zn-Mo DAs (plus NIR). Another three groups were carried out MB degradation experiments under the same conditions without the addition of BQ. Finally, the UV-vis absorbance spectra of MB were tested at 664 nm.

*1.9. Ability of Janus Zn-Mo DAs in capturing NIR-II light*

Janus Zn-Mo DAs were dispersed in aqueous solution at different concentrations (125, 250, 500, 1000 μg mL^-1^). The ability of such Janus Zn-Mo DAs in capturing NIR-II light was verified by UV-vis-NIR spectrometry.

*1.10. Photothermal stability of Janus Zn-Mo DAs*

A given Janus Zn-Mo DAs concentration (1 mg mL^-1^) was irradiated for five cycles, each composed of a 600 s heating period and a natural cooling period.

*1.11**. Calculation of photothermal conversion efficiency (η_T_) of Janus Zn-Mo DAs*

Photothermal conversion efficiency of the Janus Zn-Mo DAs was calculated by recording the change in the temperature of the NPs aqueous dispersion as a function of time under continuous irradiation for 600 s until the solution reached a steady-state temperature. The photothermal conversion efficiency (*η_T_*) was calculated according to Equation (6):

*η* =$\frac{hS\left( T_{max} - T_{surr} \right) - Q_{dis}}{I(1-{10}^{-A1064})}$ (6)

Where, *h* represents the heat transfer coefficient, *S* is the surface area of the container, *T_max_* represents the maximum steady-state temperature (66.9 °C), *T_surr_* is the ambient temperature of the environment (27.8 °C), *Q_dis_* represents the heat dissipation from the light absorbed by the solvent and the quartz sample cell, *I* is the incident laser power (1.0 W cm^-2^), and *A*^1064^ is the absorbance of the sample at 1064 nm (0.771). The value of *hS* is derived from Equation (7):

*τ* =$\frac{\boldsymbol{M}_{\boldsymbol{D}}\boldsymbol{C}_{\boldsymbol{D}}}{\boldsymbol{hS}}$ (7)

Where *τ* is the time constant for heat transfer of the system which was determined to be *τ* = 253.97 from Fig. 2f. *M_D_* and *C_D_* are respectively the mass (1 g) and heat capacity (4.2 J g^-1^) of the deionized water used to disperse the Janus Zn-Mo DAs. So the *hS* was determined to be 0.016 W. *Q_dis_* represents the heat dissipation from the light absorbed by the water and the quartz sample cell, so *Q_dis_* was calculated according to Equation (8):

*Q_dis_* =$\frac{\boldsymbol{M}_{\boldsymbol{D}}\boldsymbol{C}_{\boldsymbol{D}}\left( \boldsymbol{T}_{\mathbf{max}(\boldsymbol{water})} - \boldsymbol{T}_{\boldsymbol{surr}} \right)}{\boldsymbol{\tau}(\boldsymbol{water})}$ (8)

Where *τ*(water) is 476.2, so *Qdis* was calculated to be 0.096 W. According to the obtained data and Equation (6), the photothermal conversion efficiency of the Janus Zn-Mo DAs was determined to be 47.2%.

*1.12. Measurement of activation energy (E_a_)*

The activation energies (*E*_a_) for Zn SAs and Janus Zn-Mo DAs according to the Arrhenius equation: ln*k* = -*E*_a_/RT + lnN, where N is the pre-exponential factor, T is the absolute temperature, and R is the universal gas constant (8.314 J mol^-1^ K^-1^). Regarding the determination of the *k* value, we obtained it from the following formula: ln*c* = -*k*t + q, where q is a constant, *c* represents the concentration of H_2_O_2_, and t is the time. The rate constant *k* could be obtained from the slope of the linear plot of ln*c* vs. t.

*1.13. Cell culture*

L929 fibroblast cells and HeLa cancer cells were incubated in DMEM medium containing 10% FBS and 1% antibiotics at 37 °C under 5% CO_2_.

*1.14. Intracellular ROS measurement*

DCFH-DA was employed to detect the intracellular ROS generation ability of the Janus HZn-Mo DAs. First of all, HeLa cells were seeded onto 6-well plates and incubated for 24 h. Then, these HeLa cells can be divided into six groups including control, 1064 nm laser irradiation (NIR), HMo-ZIF8 (1 mg mL^-1^), HZn SAs (1 mg mL^-1^), Janus HZn-Mo DAs (1 mg mL^-1^), Janus HZn-Mo DAs (1 mg mL^-1^) plus NIR. After incubation for 4 h, DCFH-DA (1 mL) was added into above 6 groups, respectively. Then, the six groups of HeLa cells have be incubated in the dark for another 10 min and washed three times with PBS.

*1.15. In vitro cytotoxicity*

A typical MTT method was chosen to assess the *in vitro* cytotoxicity of the as-prepared sample. To obtain monolayer HeLa cells, these were seeded in a 96-well plate and cultured under the condition of 37 °C and 5% CO_2_ for 24 h. Eight wells of cells were left without treatment as a control group and 1064 nm laser irradiation group. Janus HZn-Mo solutions with concentrations of 15.63, 31.25, 62.5, 125, 250 and 500 μg mL^-1^ were diluted by culture medium. The other three groups of cells were treated with Janus HZn-Mo plus 1064 nm laser irradiation, HMo-ZIF8 injection, and HZn-SAs, respectively. Before being irradiated by 1064 nm laser (1.0 W cm^-2^, 5 min), the samples were put into each well and incubated for a further 3 h to achieve cell uptake. Subsequently, the culture was removed, an MTT solution (20 μL, 5 mg mL^-1^) was added, and culture was incubated for another 4 h. After that, DMSO (150 μL) was mixed into the wells; meanwhile, the absorbance located at 490 nm was recorded. The cytotoxicity was indicated by the proportion of live cells in the experimental group to the control group. *In vitro* viability and safety of Janus HZn-Mo DAs were also assessed by the MTT method using L929 fibroblast cells.

Also, calcein-AM/propidium iodide (PI) staining method was employed to further evaluate the cytotoxicity of Janus HZn-Mo DAs. HeLa cells were seeded in 6-well plates divided into six groups. The blank group received no treatment, other five groups were treated by 1064 nm laser irradiation (NIR), HMo-ZIF8, HZn SAs, Janus HZn-Mo DAs and Janus HZn-Mo DAs plus NIR, respectively. After 24 h incubation, samples were co-stained by calcein-AM/PI and subsequently imaged by CLSM.

*1.16. Flow cytometric analysis of apoptosis*

Apoptosis was analyzed using FITC/PI double staining. HeLa cells were seeded in 6-well plates and incubated for 24 h. The blank group received no treatment, other five groups were treated by 1064 nm laser irradiation (NIR) (1.0 W cm^-2^, 10 min), HMo-ZIF8, HZn SAs, Janus HZn-Mo DAs and Janus HZn-Mo DAs plus NIR (1.0 W cm^-2^, 10 min), respectively. The concentration for all nanozymes was 200 μg mL^-1^, the incubation time was 4 h. After the above six groups of treatments, the media were drained when the incubation was complete, and the cells were washed three times with PBS and subsequently detached using non–EDTA-Na–containing trypsin. The detached cells were purified twice by repetitive washing and centrifugation. The cell apoptosis was investigated by flow cytometry using the Annexin V/PI cell assay kit *via* the protocol provided by the manufacturer. The number of cells analyzed for apoptosis by flow cytometry was approximately 10,000, and the gating was also unified.

*1.17. Evaluation of mitochondrial membrane potential*

HeLa cells were seeded into 6-well plates and cultured for 24 h. control, 1064 nm laser irradiation (NIR), HMo-ZIF8 (1 mg mL^-1^), HZn SAs (1 mg mL^-1^), Janus HZn-Mo DAs (1 mg mL^-1^), Janus HZn-Mo DAs (1 mg mL^-1^) plus NIR for 4 h, respectively. After washing with PBS, the cells were stained with JC-1 and detected by CLSM.

*1.18. Biological transmission electron microscopy (Bio-TEM)*

The monolayer HeLa cells were cultured with 1 mL of cultural solution containing Janus HZn-Mo DAs (1 mg) for 1 h. Then, the cells were rinsed 3 times with PBS and further fixed for more than 24 h. Finally, the TEM images of these cells were observed by biological transmission electron microscopy.

*1.19. Western blot*

HeLa cells were preseeded in a six-well culture plate (1 × 10^5^ cells per well). For HIF-1α assays, the experiment was divided into two groups: (i) control, (ii) Janus HZn-Mo DAs. The HeLa cells were collected using trypsin and lysed in a lysis buffer. Then, the proteins were separated by SDS- polyacrylamide gel electrophoresis and then transferred into a polyvinylidene fluoride membrane. After blocking with 5% dried skimmed milk for 1 h, the samples were stained with the corresponding primary antibody overnight and anti-β-actin antibody as the loading control, followed by cultivation with the goat anti-rabbit Dylight-800 antibody for 1 h. Afterward, the membrane was visualized by an Odyssey CLx Image Studio.

*1.20. Antitumor study*

Female Kunming mice (20-22 g, aged about 6 weeks) were obtained from Second Affiliated Hospital of Harbin Medical University (Harbin, China) and all animal experiments were approved by the Laboratory Animal Management and Ethics Committee of Northeast Forestry University. Animal experimental procedures were executed in accordance with the Guidelines for Care and Use of Laboratory Animal Management and Ethics Committee of Northeast Forestry University (No. 2025019). Under sterile conditions, 7- to 10-day-old ascites of uterine cervical carcinoma (U14) cells were collected from the mice and diluted to 1 × 10^7^ cells mL^-1^ with sterile physiological saline. Typically, Female Kunming mice were subcutaneously transplanted with U14 cancer cells (100 µL, 1 × 10^7^ cells mL^-1^). After the tumor size increased approximately 120 mm^3^, the mice were randomly divided into 6 groups and treated with PBS injection (control, group 1), PBS injection plus 1064 nm laser irradiation (group 2), HMo-ZIF8 injection (group 3), HZn-SAs injection (group 4), Janus HZn-Mo DAs injection (group 5), and Janus HZn-Mo DAs plus 1064 nm laser irradiation (1 W cm^-2^) for 10 min (group 6), respectively. The body weight and tumor size were recorded every 2 days after the first treatment. The tumor volume (mm^3^) of mice was calculated by the formula *V* = *lw*^2^/2, in which *l* and *w* are the length and width of the tumor. The nanozymes were administered *via* intravenous injection (tail vein) at a dose of 100 μL (200 μg mL^-1^).

*1.21. Blood routines and serum biochemistry assay*

Ten healthy Kunming female mice were divided into two groups (n = 5). One was used as a control group, and the other group was injected with 50 μL PBS solution containing 500 µg mL^-1^ of Janus HZn-Mo DAs. After 7 and 14 days, the blood samples were obtained by extracting eyeball blood to test the blood routines and serum biochemistry indicators.

*1.22. Hemolysis test*

Hemolysis assay was performed by dispersing different concentrations of Janus HZn-Mo DAs (25, 50, 100 and 200 µg mL^-1^) in PBS and then the suspension was added to the red blood cells of mice. H_2_O and PBS were used as positive and negative controls, respectively. The mixture was allowed to stand at room temperature for 4 h and the supernatant was collected by centrifugation at 3500 rpm for 5 min. The absorbance at 540 nm was then measured and the hemolytic ratio was calculated by Equation (9):

Hemolysis = $\frac{\boldsymbol{A}_{\boldsymbol{sample}}\boldsymbol{-}\boldsymbol{A}_{\boldsymbol{negative}}}{\boldsymbol{A}_{\boldsymbol{positive}}\boldsymbol{-}\boldsymbol{A}_{\boldsymbol{negative}}}$ (9)

*1.23. Histological examination*

After 14 days of treatment, the heart, liver, spleen, lung, kidney, and tumors less than 1 cm × 1 cm in control, 1064 nm laser irradiation, HMo-ZIF8, HZn-SAs, Janus HZn-Mo DAs and Janus HZn-Mo DAs plus 1064 nm laser irradiation treated mice were excised and dehydrated with buffered formalin, ethanol, and xylene in turn. Finally, all types of dehydrated tissues were embedded in liquid paraffin to obtain stained slices for H&E, Ki67 and Tunel staining by optical microscope.

*1.24. Biodistribution*

The mice were injected intravenously with Janus Zn-Mo DAs or Janus HZn-Mo DAs, then were euthanized at time points of 0 h, 6 h, 12 h and 24 h. The collected major tissues (heart, liver, spleen, lung, kidney, and tumor) were dissolved in aqua regia. To obtain clear solutions, the mixture was heated to 70 °C, maintained for 5 min, and centrifuged for further ICP-OES analysis.

*1.25. Statistical analysis*

All data in this work were shown as means ± SD. Unless otherwise mentioned, all statistical analyses were conducted by using GraphPad Prism 9.5. The difference between two groups was performed using Student’s t test, and multiple group comparisons were analyzed by one-way ANOVA with a Bonferroni post hoc test for comparisons. n.s. represents no difference. **p* < 0.05, ***p* < 0.01, ****p* < 0.001, and *****p* < 0.0001 were considered statistically significant. Each experiment included at least three replicates.


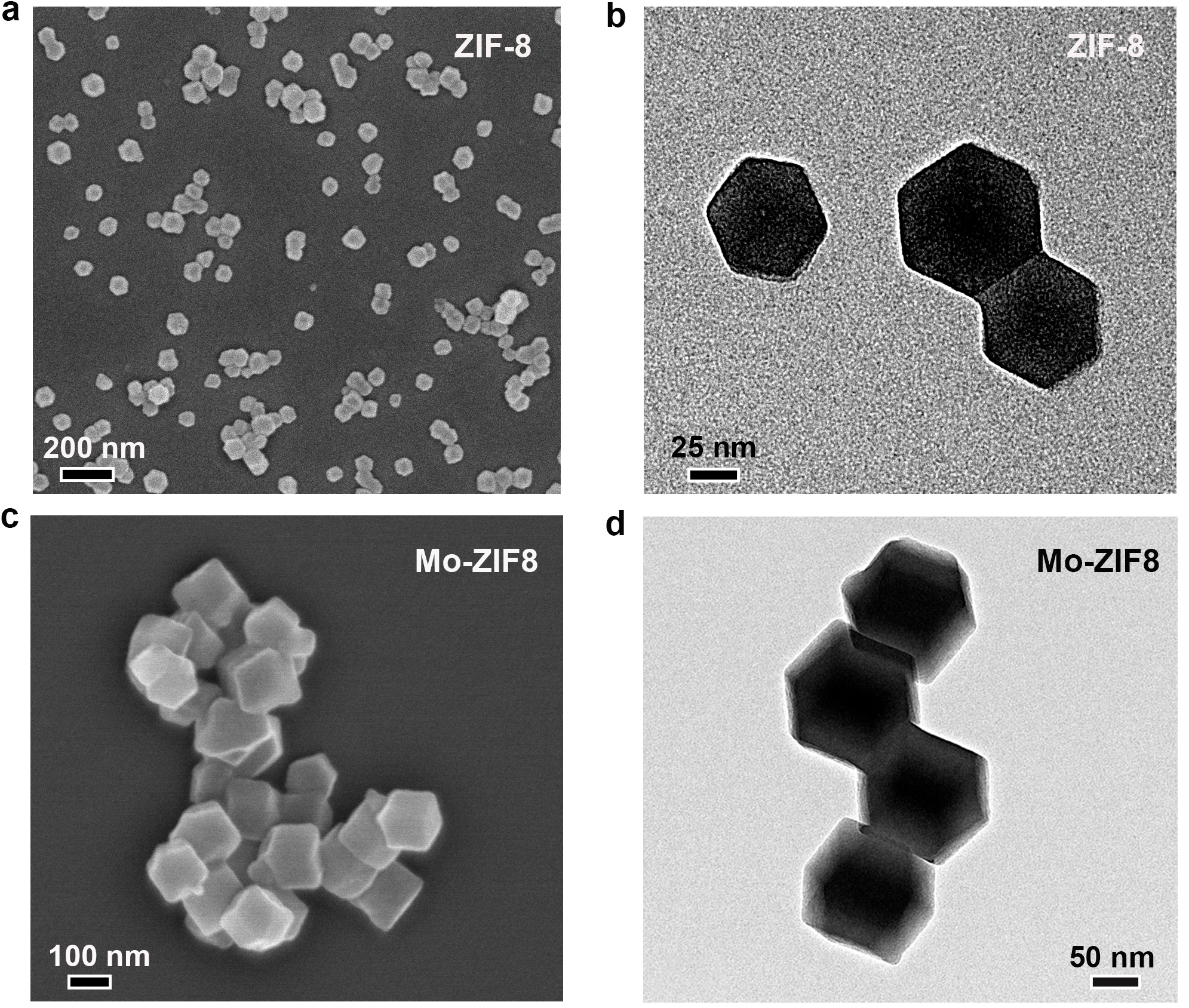


**Fig. S1.** SEM (a) and TEM (b) images of ZIF-8. SEM (c) and TEM (d) images of Mo-ZIF8.


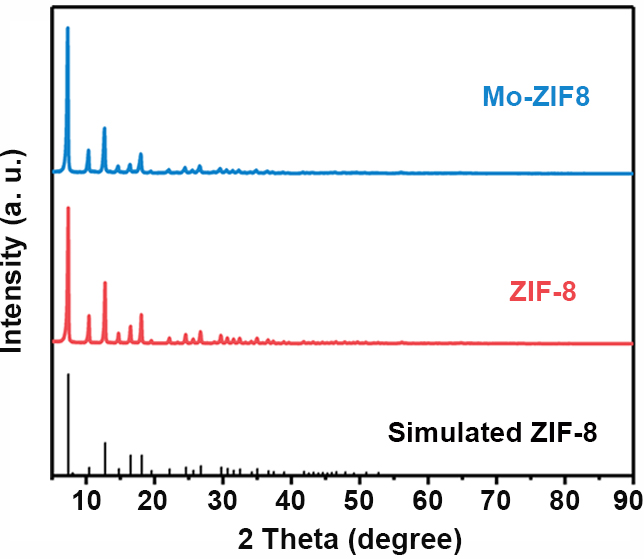


**Fig. S2**. XRD patterns of ZIF-8, Mo-ZIF8 and the simulated ZIF-8.


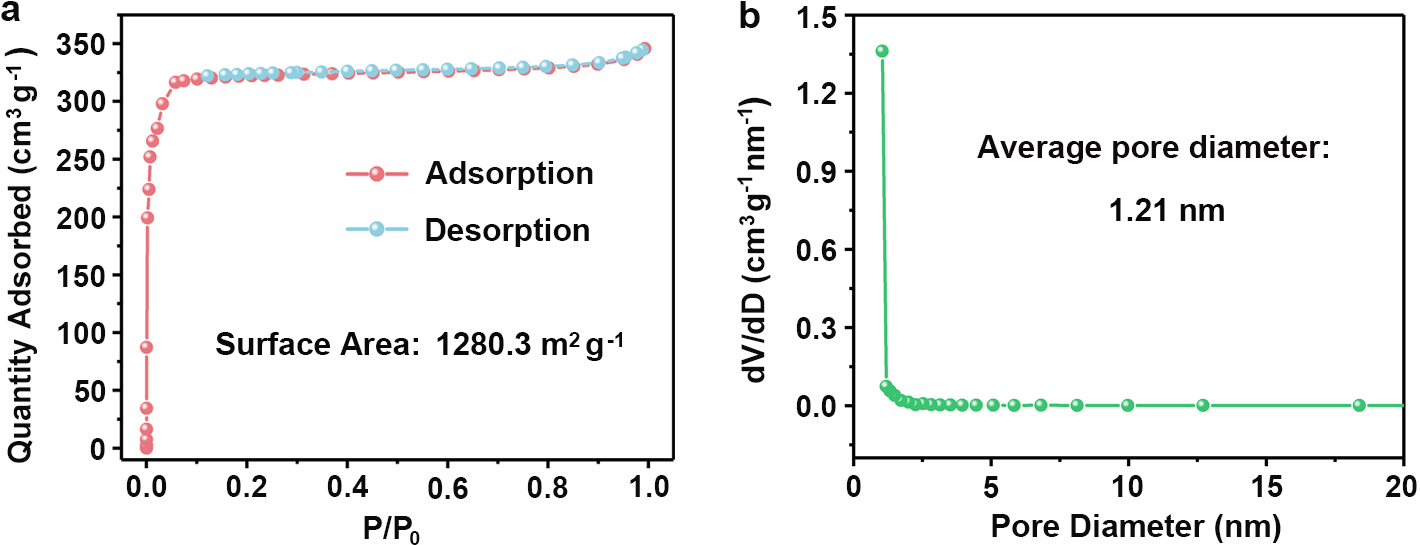


**Fig. S3**. Nitrogen absorption-desorption isotherm (a) and pore diameter distribution (b) of ZIF-8.


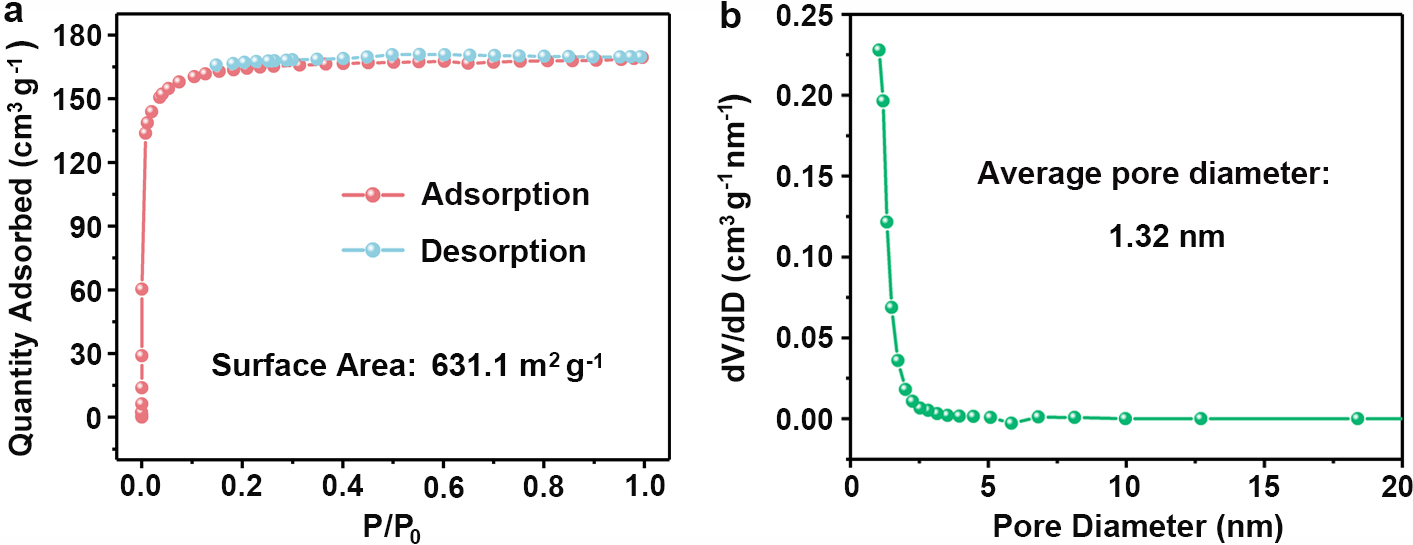


**Fig. S4**. Nitrogen absorption-desorption isotherm (a) and pore diameter distribution (b) of Janus Zn-Mo DAs.


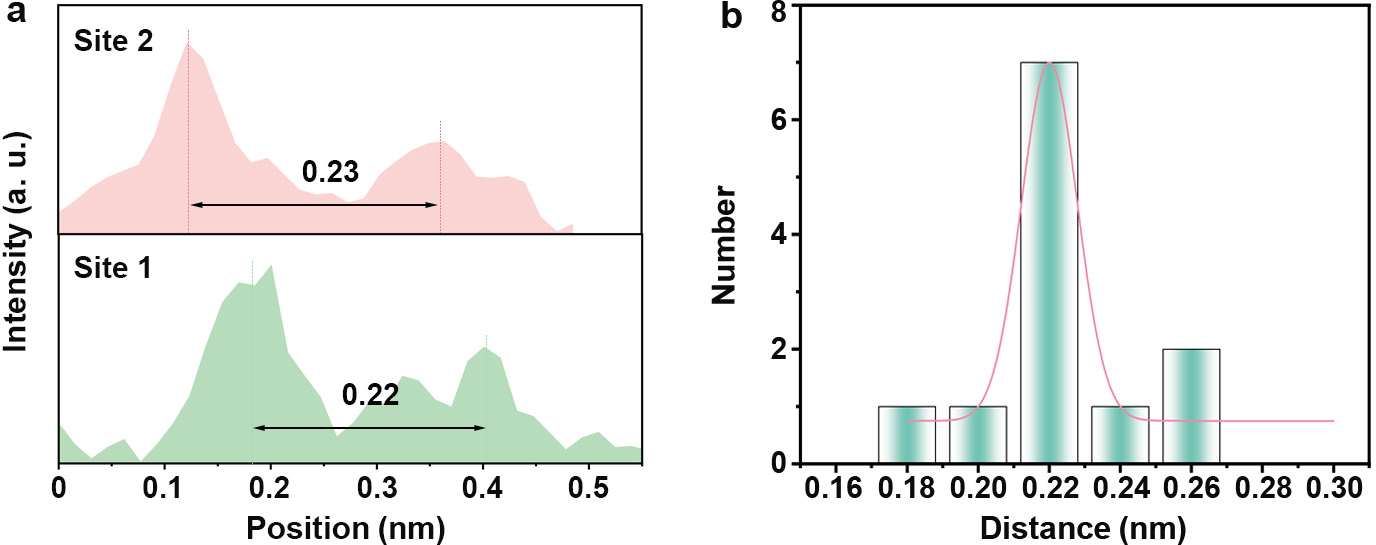


**Fig. S5.** Corresponding intensity profiles of dual atoms pair (a). Distance distribution between neighboring Zn-Mo moieties (b).


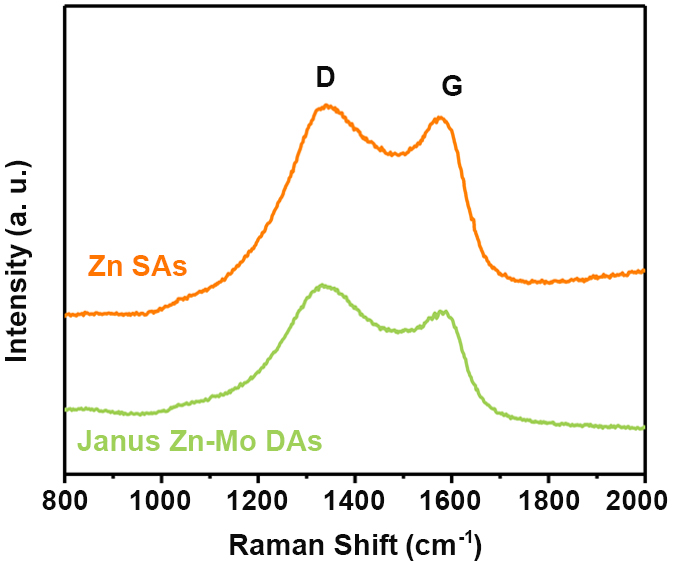


**Fig. S6**. Raman spectra of Zn SAs and Janus Zn-Mo DAs.


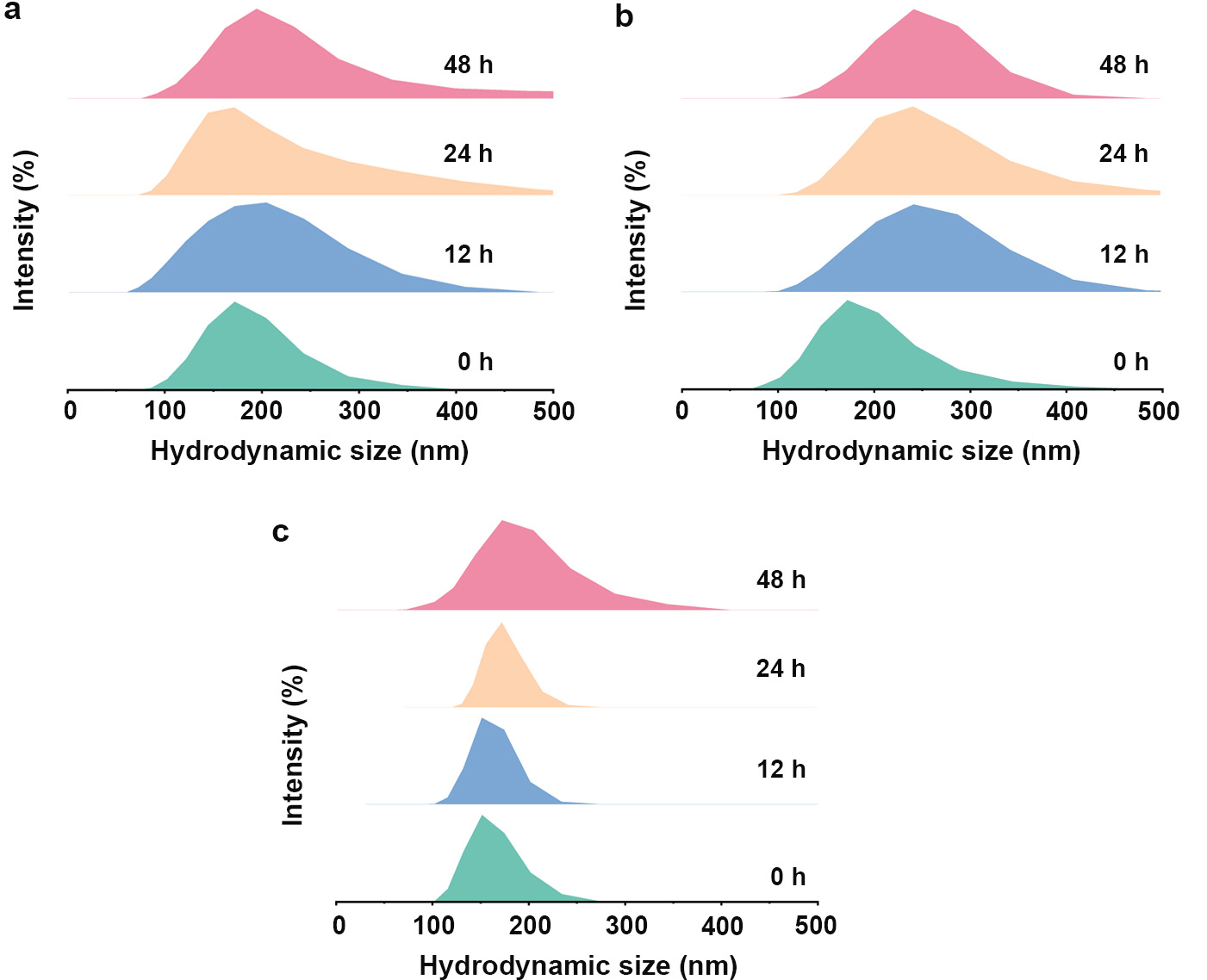


**Fig. S7**. Size distributions of Janus Zn-Mo DAs in pure PBS (a), PBS containing 10% FBS (b), and acidic PBS with GSH (c) solutions measured by DLS.


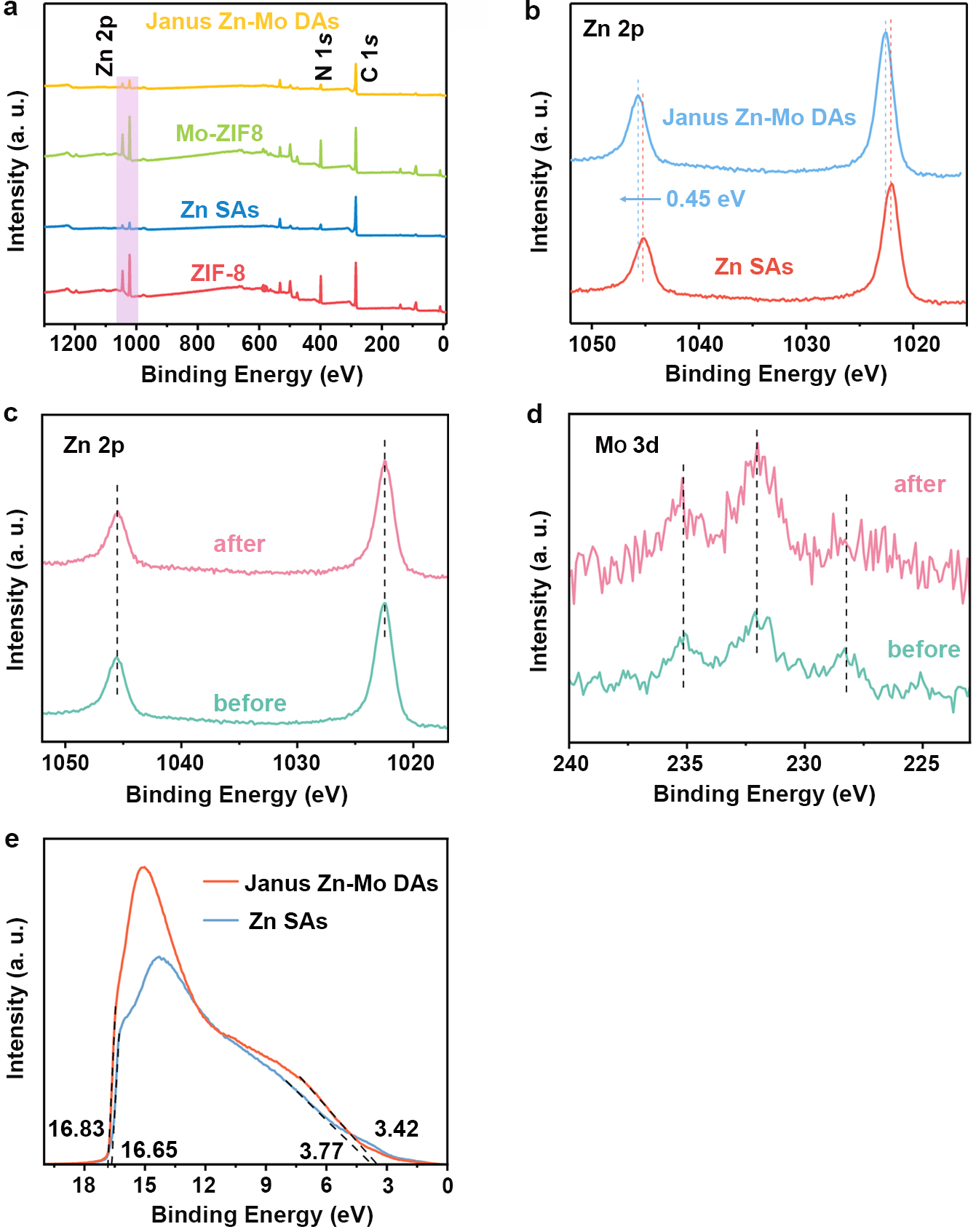


**Fig. S8**. XPS survey spectra for ZIF-8, Zn SAs, Mo-ZIF8 and Janus Zn-Mo DAs (a). High-resolution Zn 2p XPS spectra of Zn SAs and Janus Zn-Mo DAs (b). High-resolution Zn 2p (c) and Mo 3d (d) XPS spectra of Janus Zn-Mo DAs before and after incubation with simulated tumor interstitial fluid. The UPS spectra of Zn SAs and Janus Zn-Mo DAs (e).


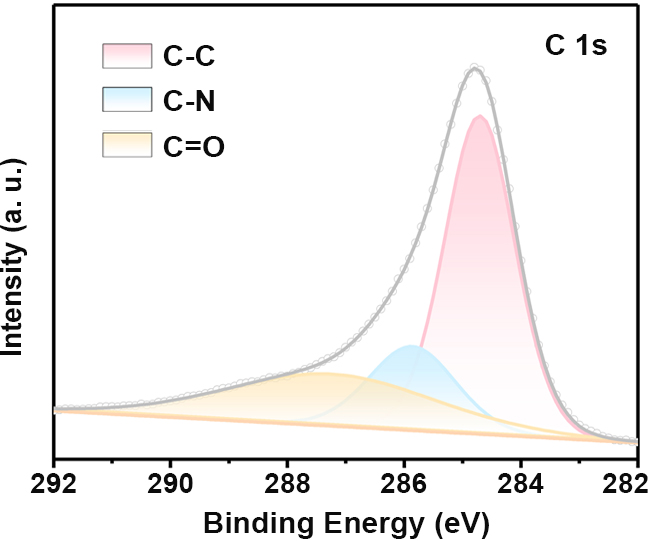


**Fig. S9**. The high-resolution C 1s XPS spectra of Janus Zn-Mo DAs.


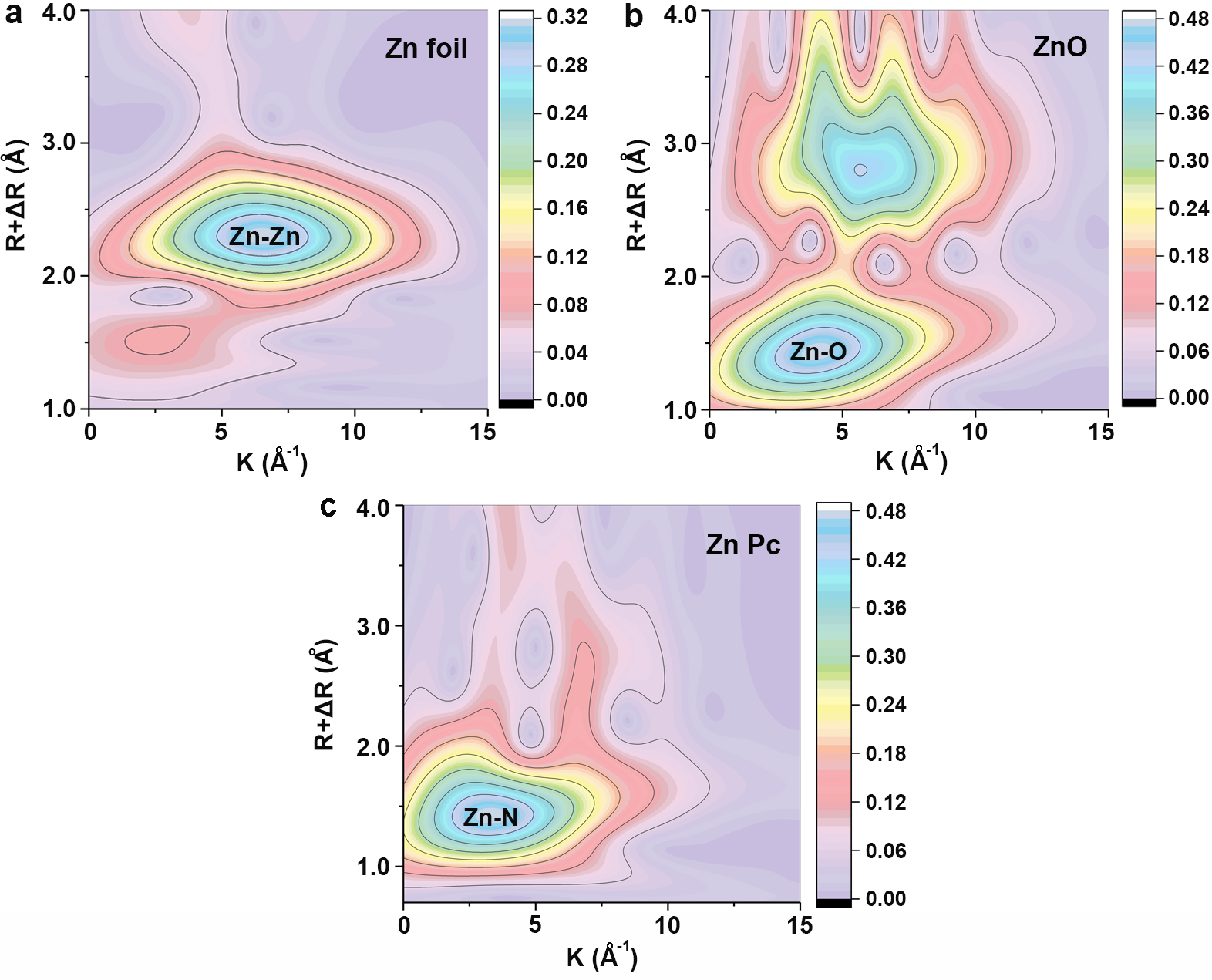


**Fig. S10**. WT-EXAFS contour maps of Zn foil (a), ZnO (b) and ZnPc (c).


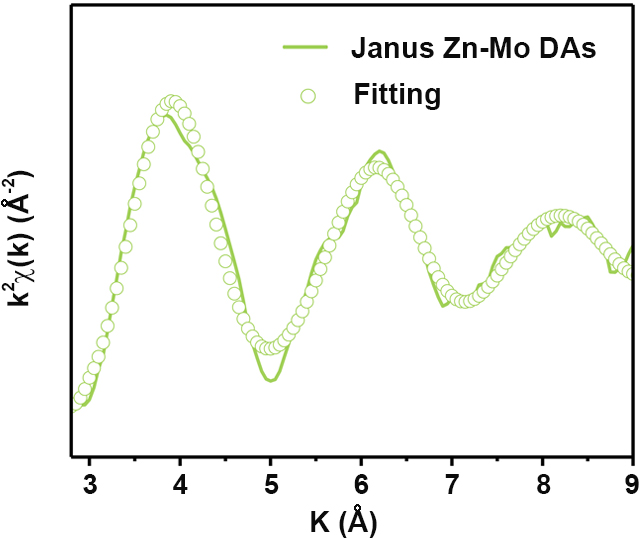


**Fig. S11**. K^2^χ(k) space spectrum fitting curve of Janus Zn-Mo DAs.


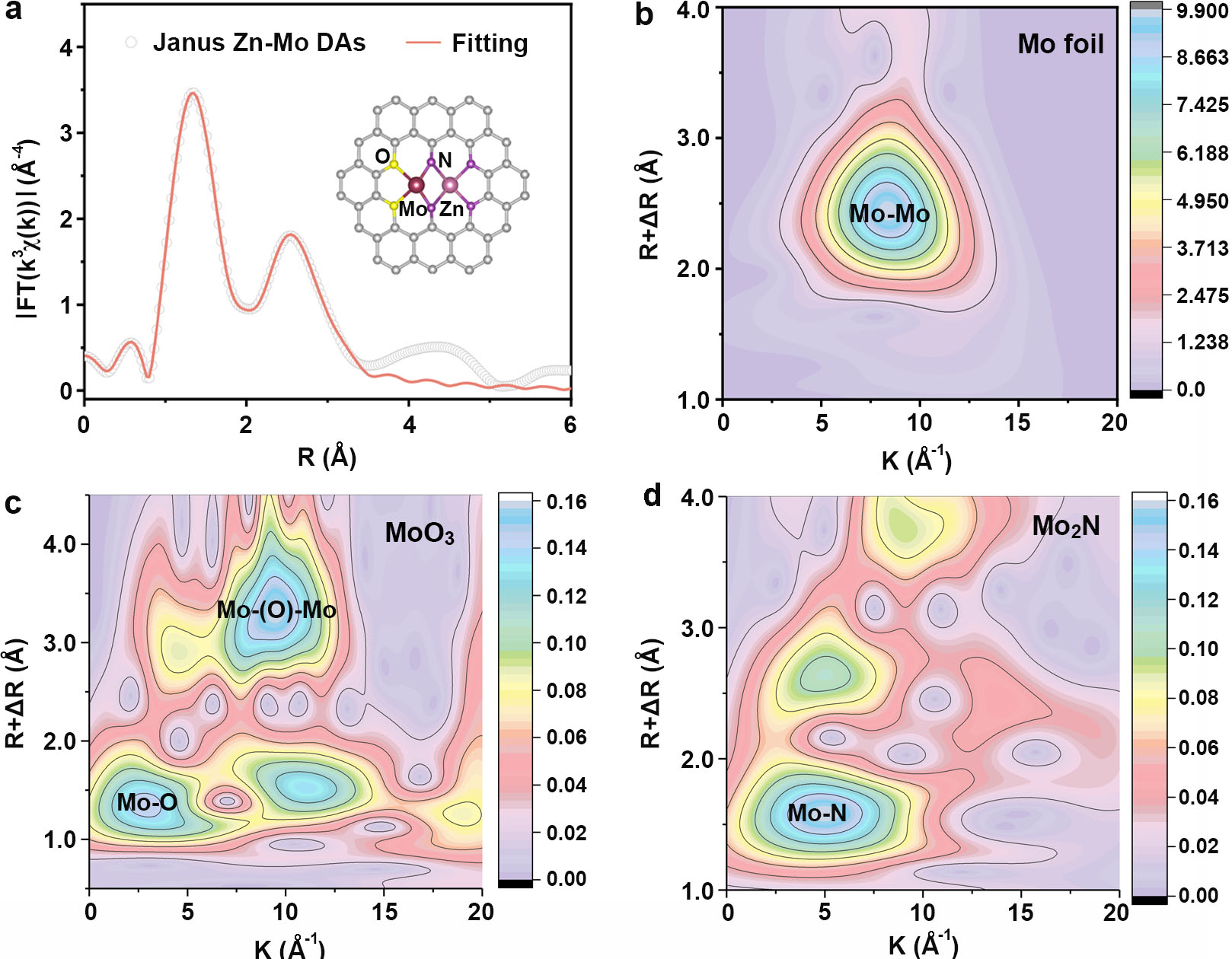


**Fig. S12.** The experimental and fitting results of the FT-EXAFS spectrum of Janus Zn-Mo DAs in *R* space. Inset: proposed schematic model (a). WT-EXAFS contour maps of Mo foil (b), MoO_3_ (c) and Mo_2_N (d).


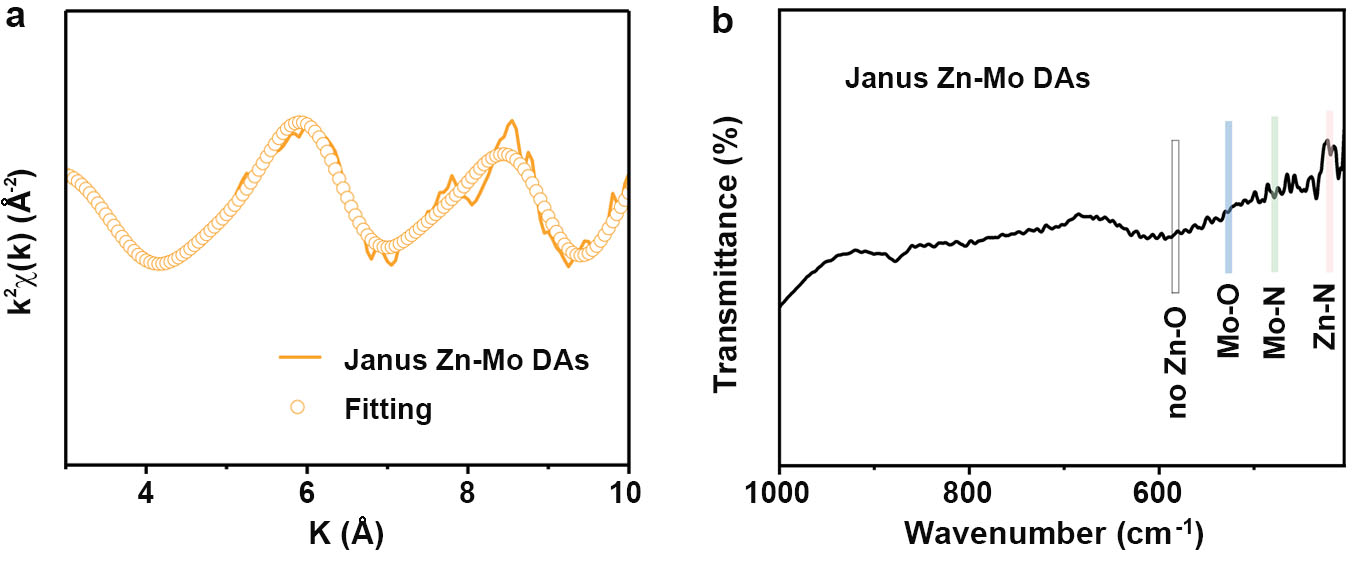


**Fig. S13.** K^2^χ(k) space spectrum fitting curve of Janus Zn-Mo DAs (a). FT-IR spectrum of Janus Zn-Mo DAs (b).


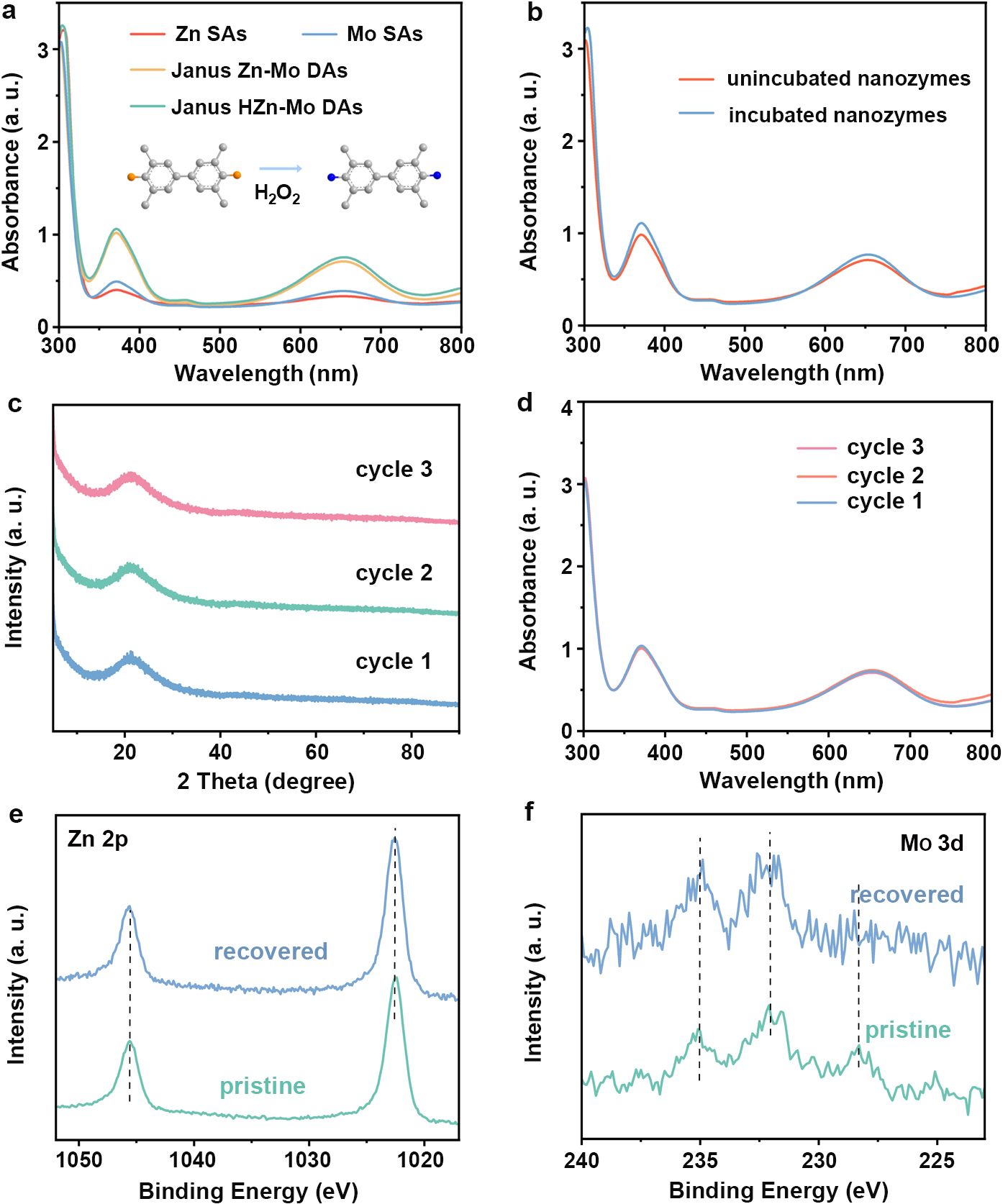


**Fig. S14.** UV-vis absorption spectra changes of the catalyzed oxidation of TMB (oxTMB) with different treatments (a). UV-vis absorption spectra of oxTMB for Janus Zn-Mo DAs before and after incubated with acidic PBS with GSH solutions (b). The XRD patterns (c) and UV-vis absorption spectra of oxTMB (d) for Janus Zn-Mo DAs after three catalytic cycles. High-resolution Zn 2p (e) and Mo 3d (f) XPS spectra of Janus Zn-Mo DAs before and after catalytic reaction.


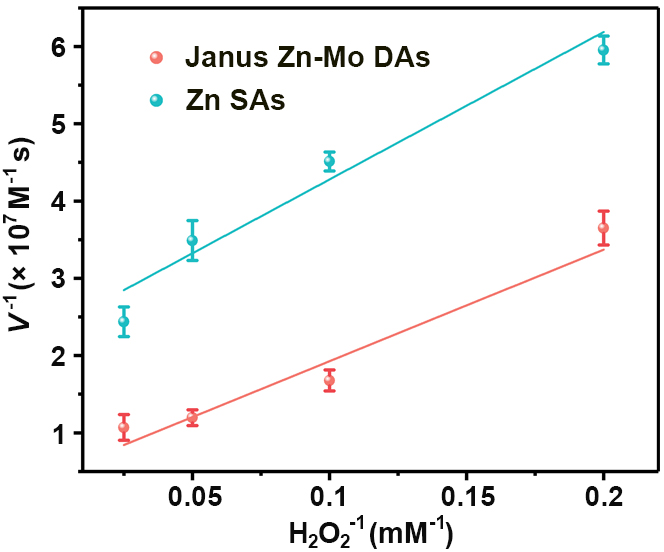


**Fig. S15.** Lineweaver-Burk plotting for Zn SAs and Janus Zn-Mo DAs with H_2_O_2_ as a substrate. Data were presented as mean ± S.D. (n = 3).


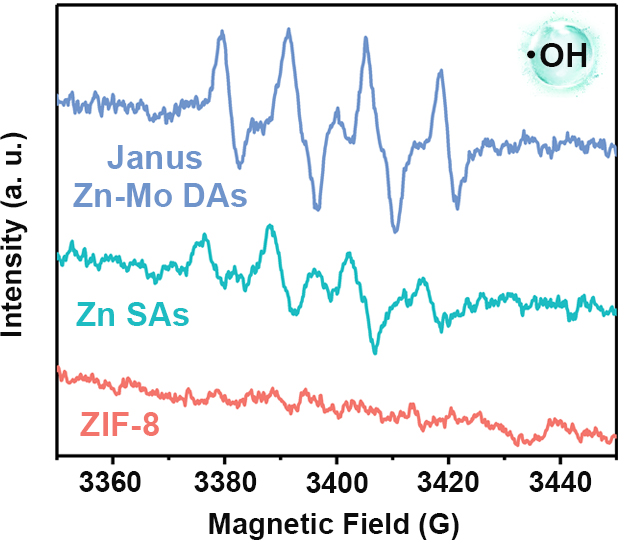


**Fig. S16.** ESR spectra of •OH generation after different conditions.


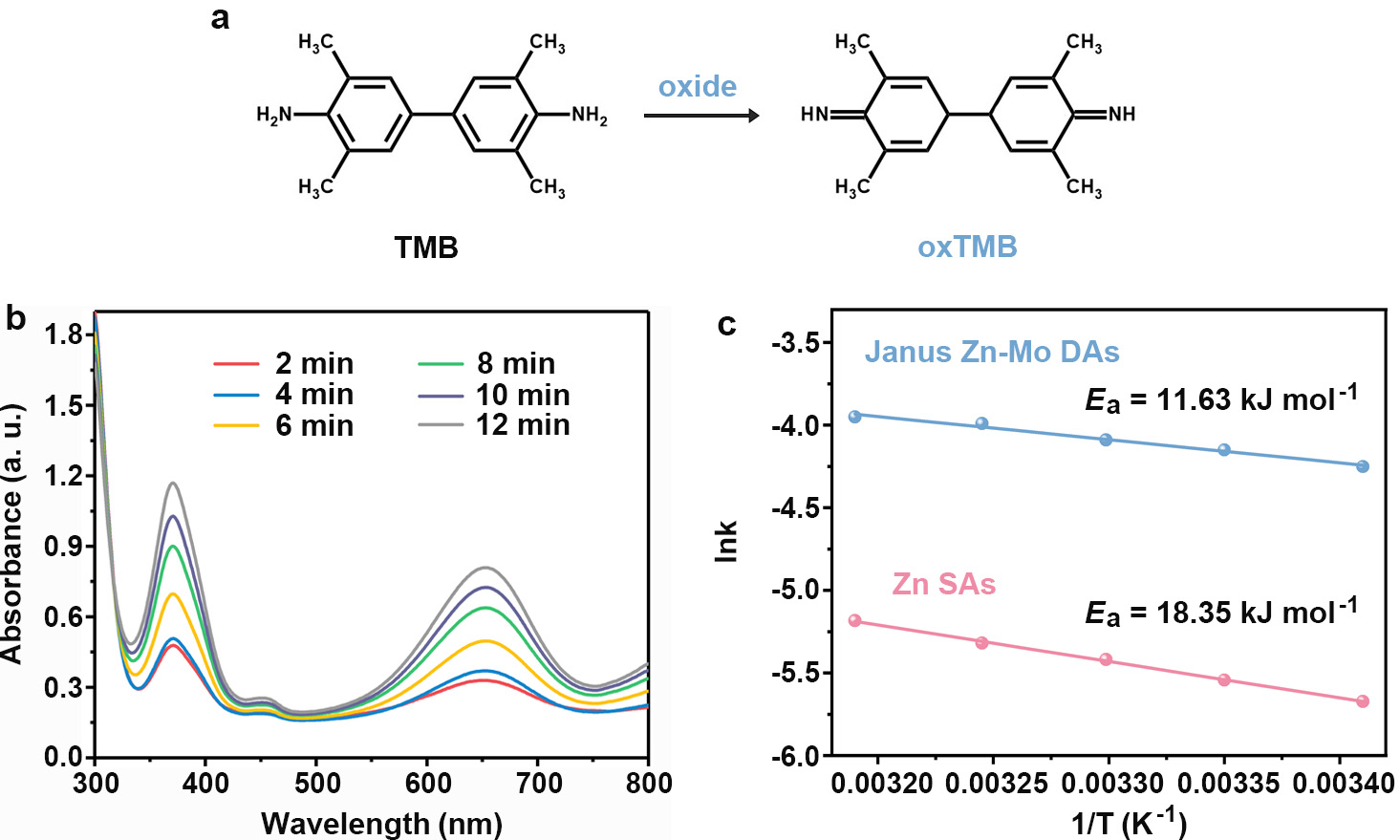


**Fig. S17.** Schematic diagram of TMB for •OH test (a). Time-dependent absorbance of TMB in the presence of H_2_O_2_ and Janus Zn-Mo DAs (b). Logarithmic reaction rate over Zn SAs and Janus Zn-Mo DAs as a function of the reciprocal temperature (c).

**
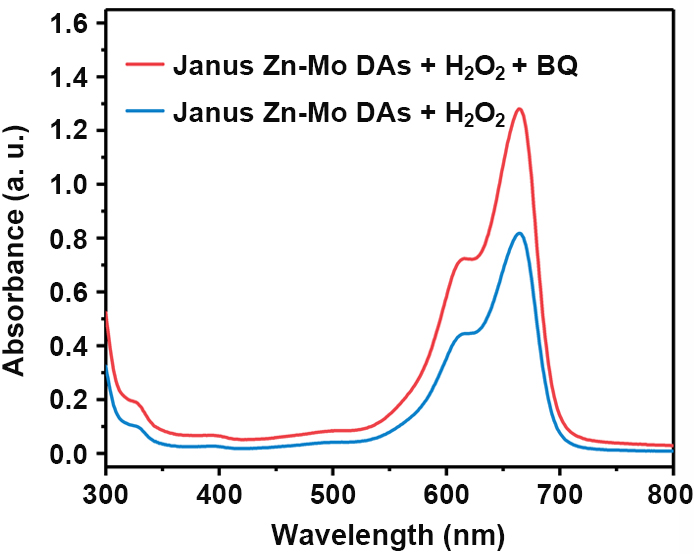
**

**Fig. S18.** UV-Vis absorption curves of MB after different treatments.


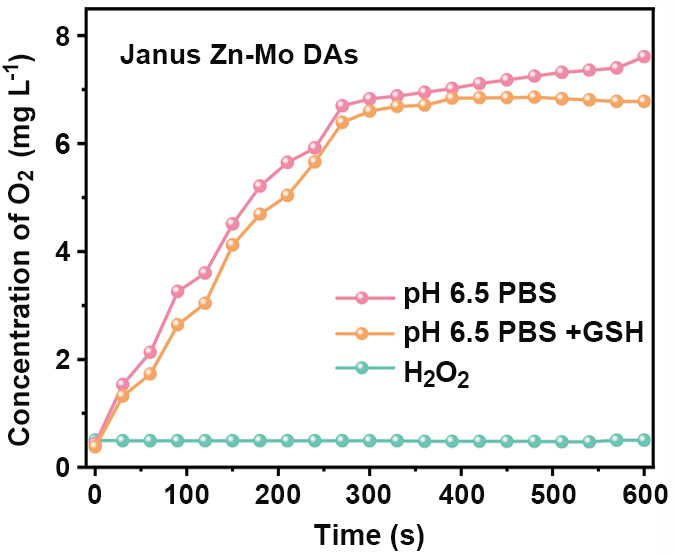


**Fig. S19.** The concentration of O_2_ in different solutions.


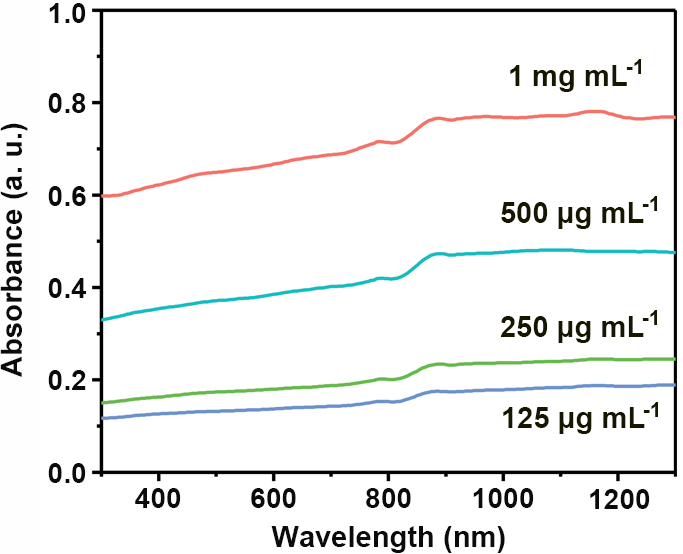


**Fig. S20.** UV-Vis-NIR absorbance spectra of the Janus Zn-Mo DAs solutions with different concentrations.


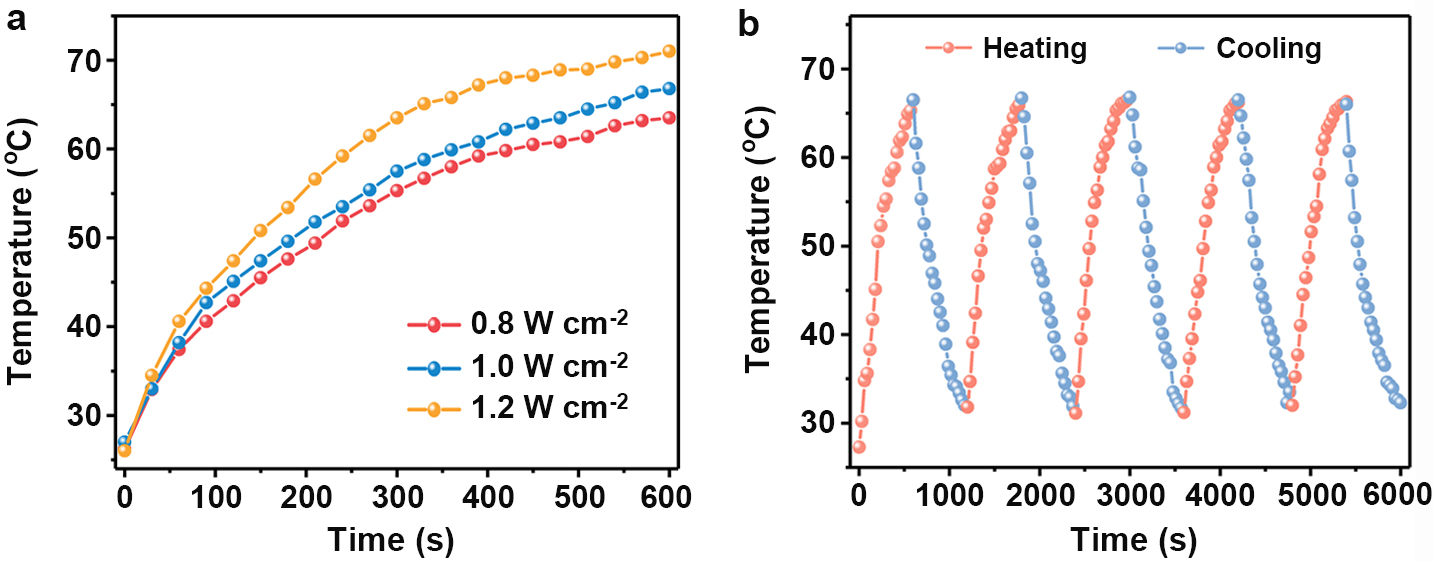


**Fig. S21**. Photothermal heating curves of dispersed Janus Zn-Mo DAs (1 mg mL^-1^) under 1064 nm laser irradiation at varied power densities (a). Photothermal stability tests of Janus Zn-Mo DAs (1 mg mL^-1^) under NIR-II laser irradiation (1.0 W cm^-2^) (b).


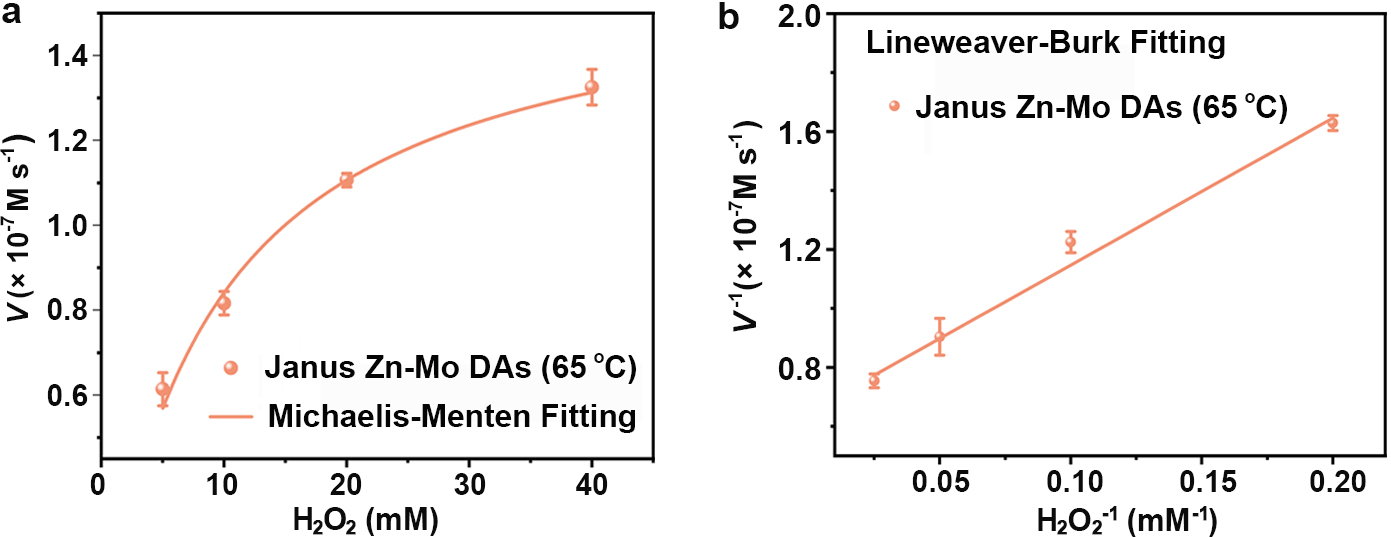


**Fig. S22.** Michaelis-Menten kinetic analysis (a) and Lineweaver-Burk plot (b) of Janus Zn-Mo DAs (65 ℃) for POD-like activities with H_2_O_2_ as the substrate. Data were presented as mean ± S.D. (n = 3).


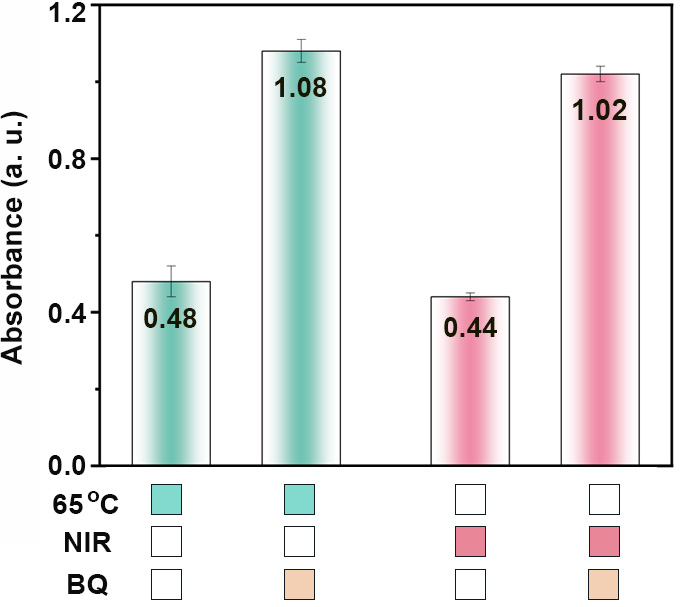


**Fig. S23.** The absorbance changes of MB aqueous solutions under different conditions. Data were presented as mean ± S.D. (n = 3).


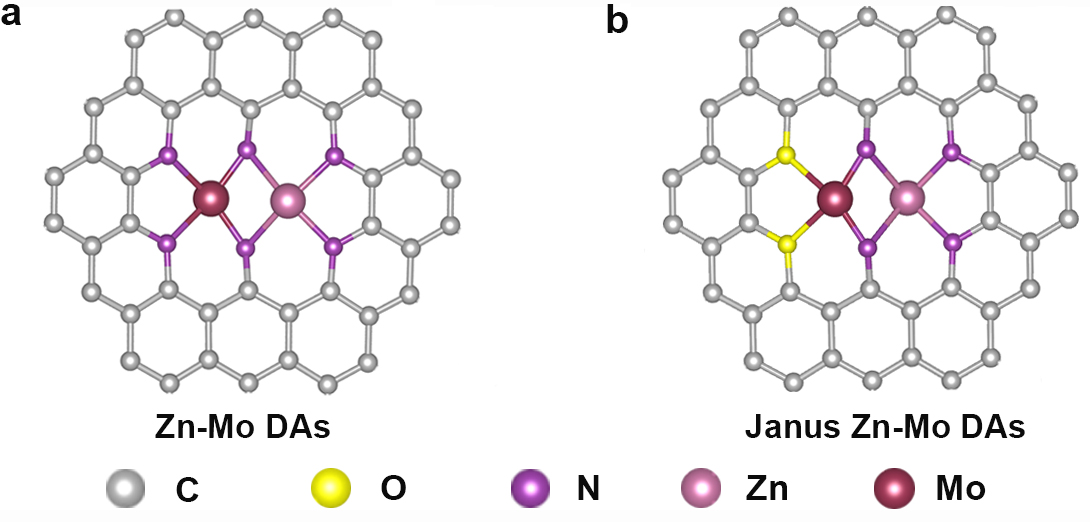


**Fig. S24.** The model of Zn-Mo DAs (a) and Janus Zn-Mo DAs (b).


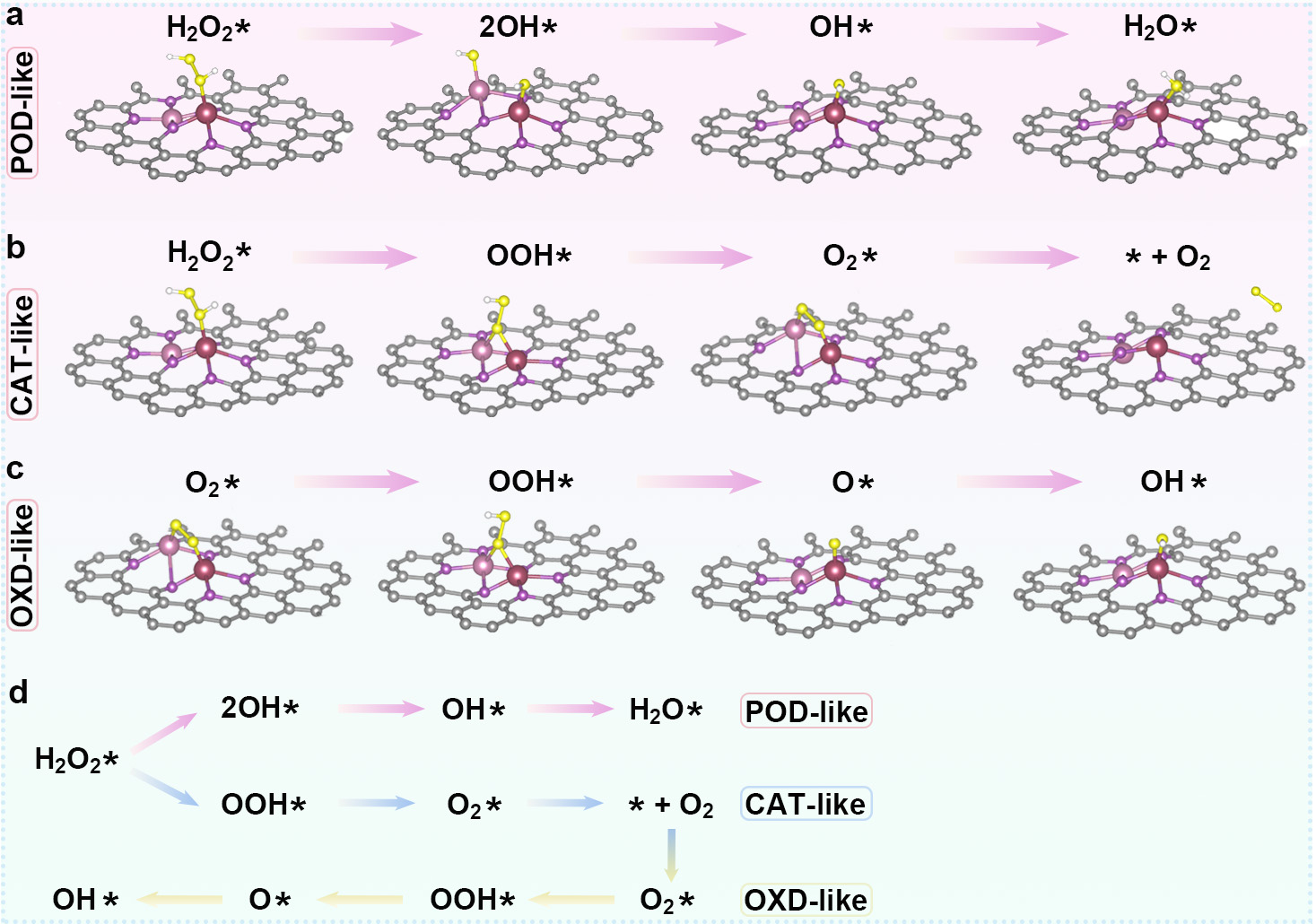


**Fig. S25.** Catalytic mechanisms along the POD-like (a), CAT-like (b) and OXD-like (c) reaction paths on the Zn-Mo DAs. Schematic illustrations of the distinct catalytic mechanisms of the POD-like, CAT-like, and OXD-like reaction paths (d).


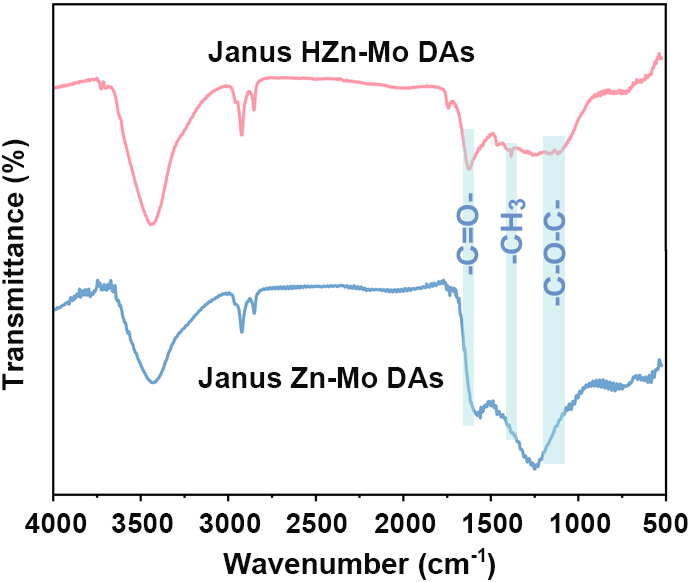


**Fig. S26.** FTIR spectra of Janus Zn-Mo DAs and Janus HZn-Mo DAs.


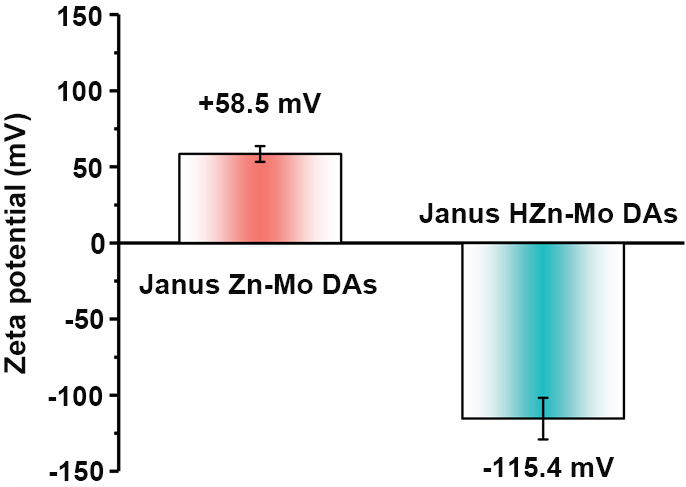


**Fig. S27.** The zeta potential testing results of Janus Zn-Mo DAs and Janus HZnMo DAs. Data were presented as mean ± S.D. (n = 3).


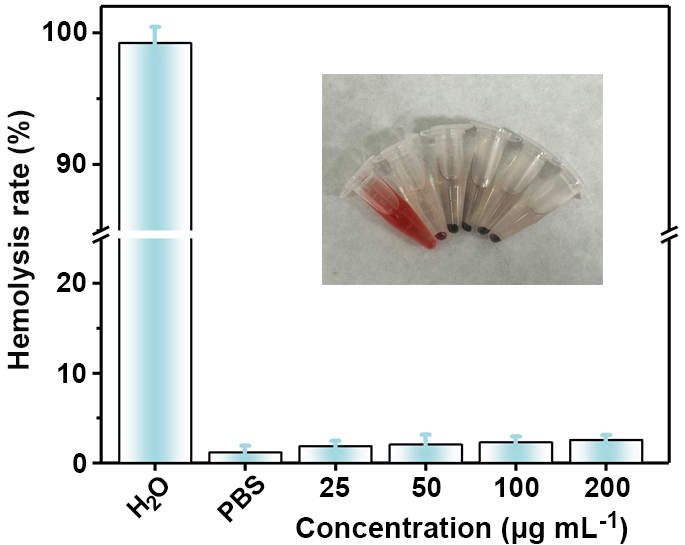


**Fig. S28.** Hemolytic assay for Janus HZn-Mo DAs with disparate concentrations. Data were presented as mean ± S.D. (n = 3).


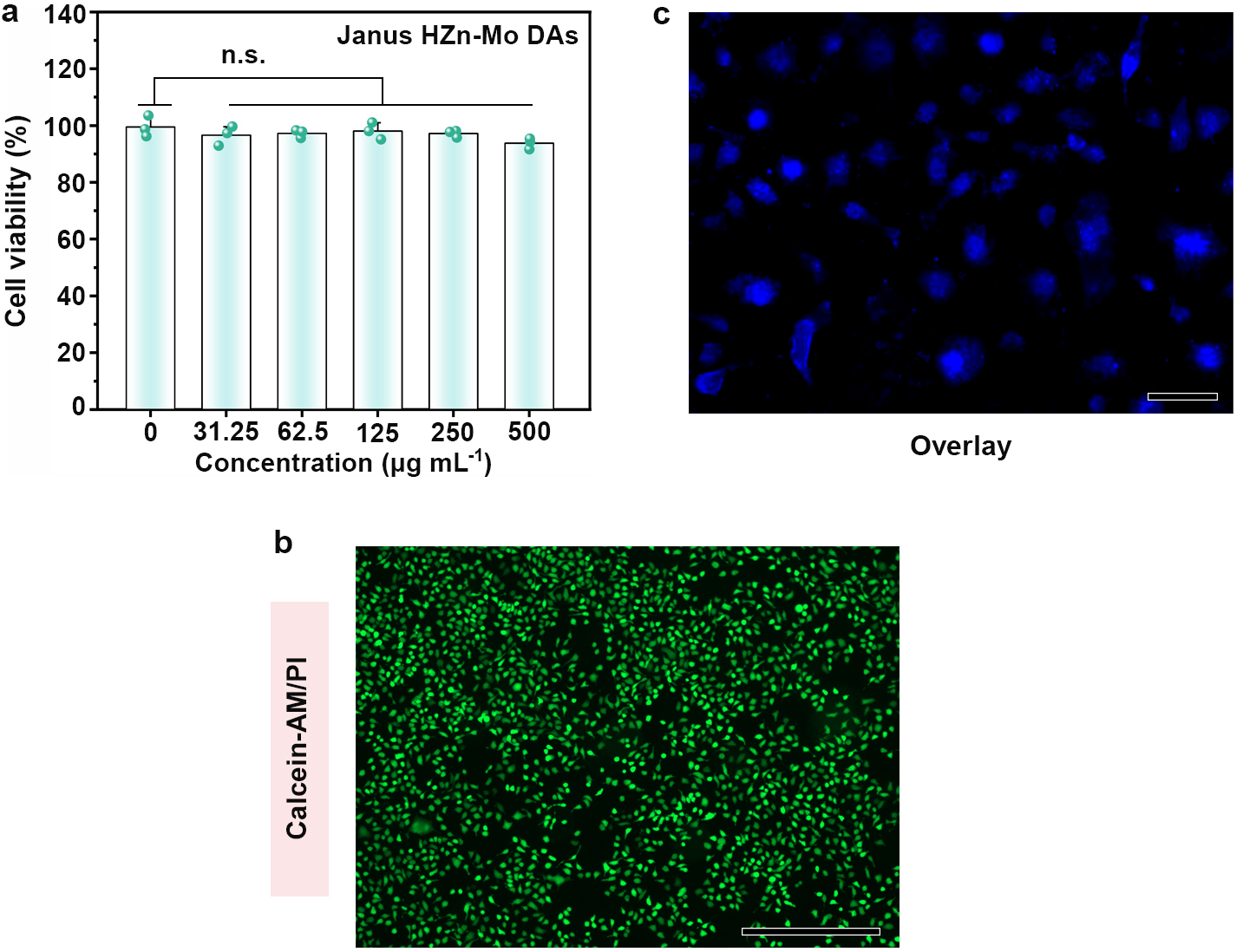


**Fig. S29.** Viabilities of L929 cells treated with Janus HZn-Mo DAs. Statistical analysis was performed *via* one-way ANOVA with a Bonferroni post hoc test for comparisons. n.s. represents no difference. Data were presented as means ± SD (n = 3) (a). CLSM image dyed with AM and PI of L929 cells in the Janus HZn-Mo DAs plus 1064 nm laser irradiation group (b). CLSM images of L929 cells after treatment with Janus HZn-Mo DAs-FITC for 1 h (c). Scale bar, 50 μm.


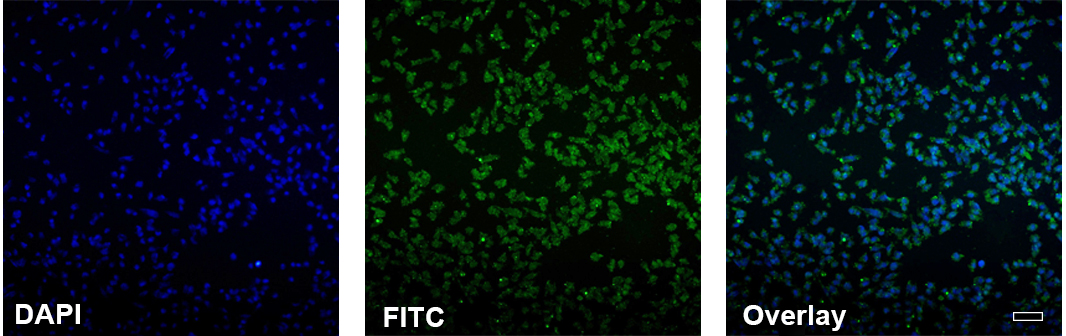


**Fig. S30.** CLSM images of HeLa cells after treatment with Janus HZn-Mo DAs-FITC for 1 h. Scale bar: 100 μm.


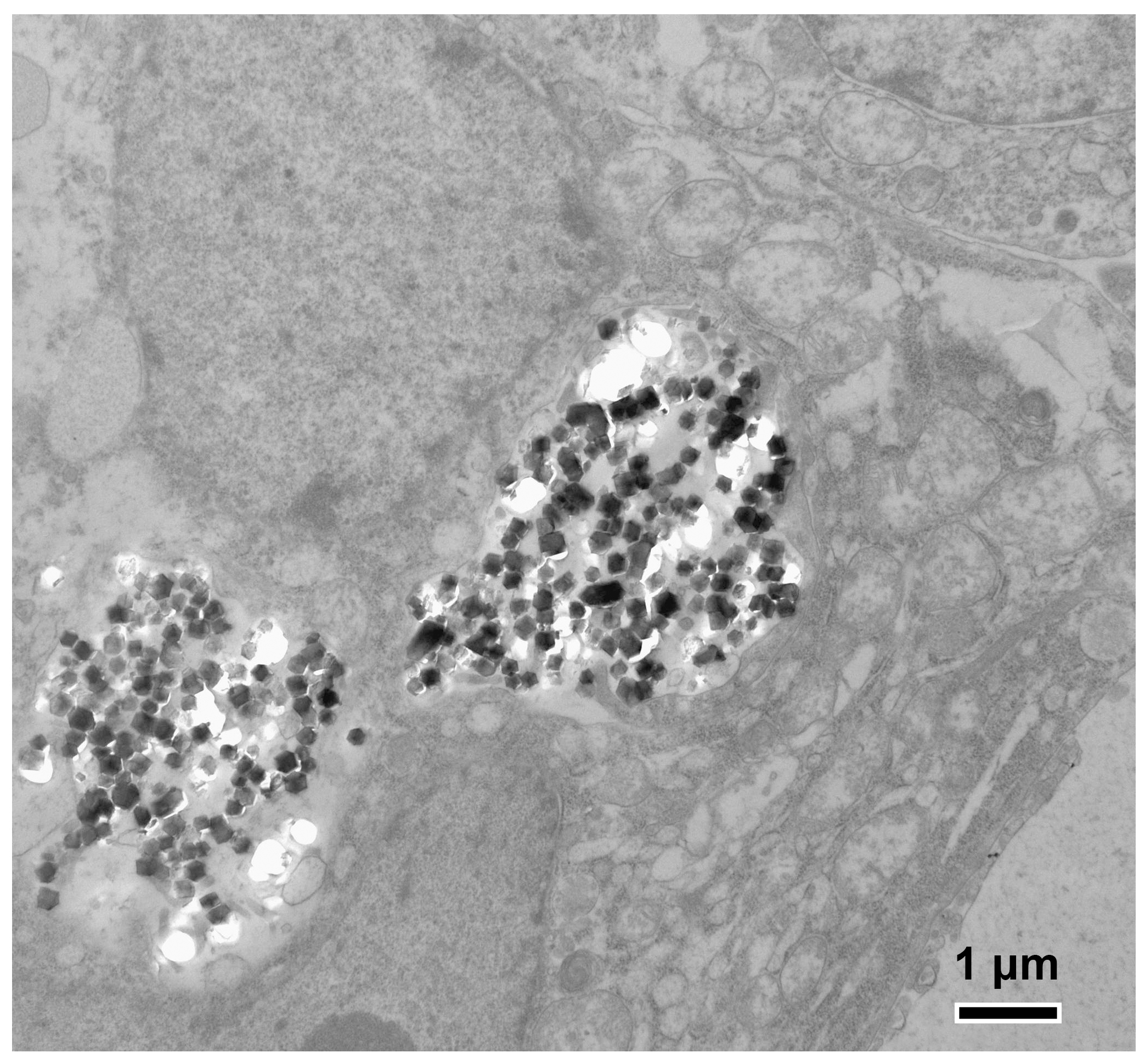


**Fig. S31.** Bio-TEM image of HeLa cells incubated with Janus HZn-Mo DAs for 1 h.


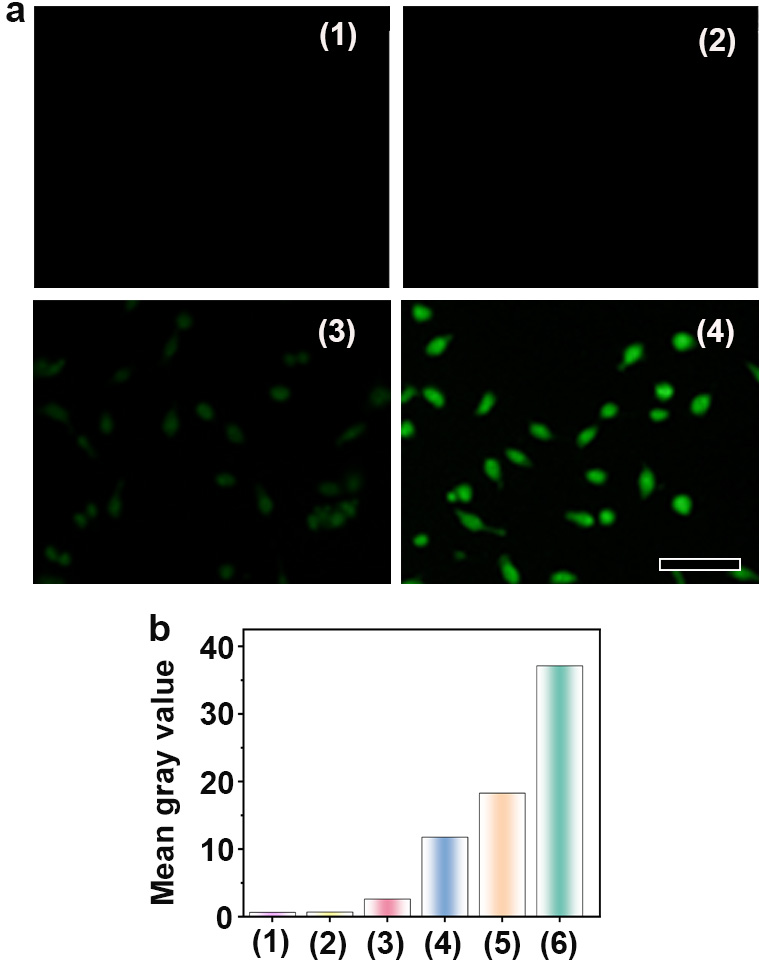


**Fig. S32.** Intracellular ROS detection using DCFH-DA probe in the (1) PBS (Control), (2) PBS plus 1064 nm (NIR), (3) HMo-ZIF8, (4) HZn SAs. Scale bar: 50 μm (a). The corresponding quantitative data of fluorescence in the (1)-(4), (5) Janus HZn-Mo DAs, or (6) Janus HZn-Mo DAs plus NIR (b).


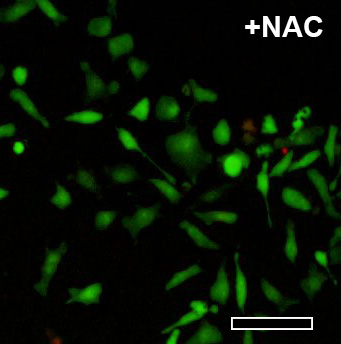


**Fig. S33.** CLSM images of HeLa cells stained with calcein-AM (green) and PI (red) after treatment with Janus HZn-Mo DAs plus NIR in the presence of NAC. Scale bar: 50 μm.


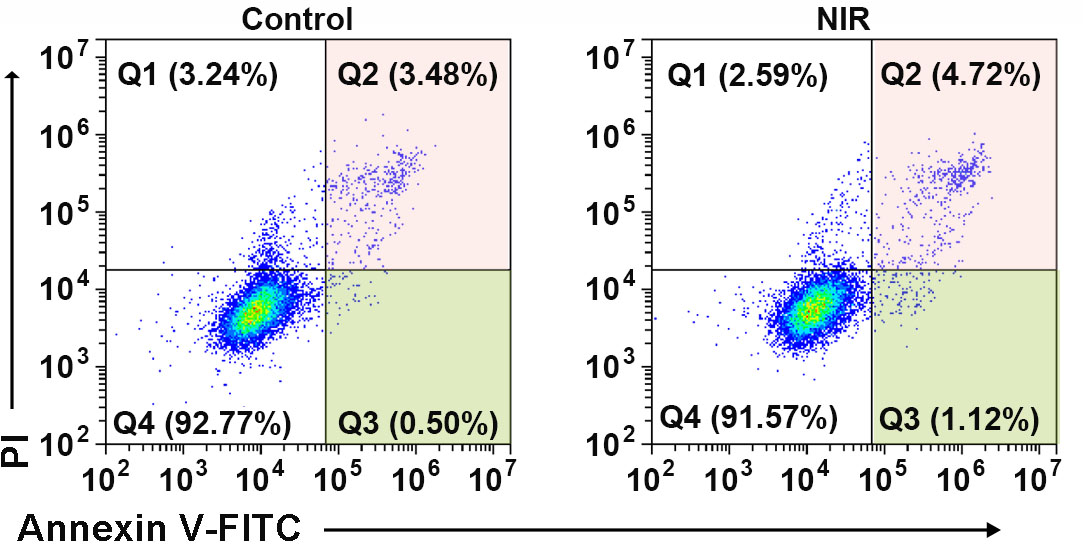


**Fig. S34.** Apoptosis of HeLa cells detected by flow-cytometry in the groups of control and NIR.

**
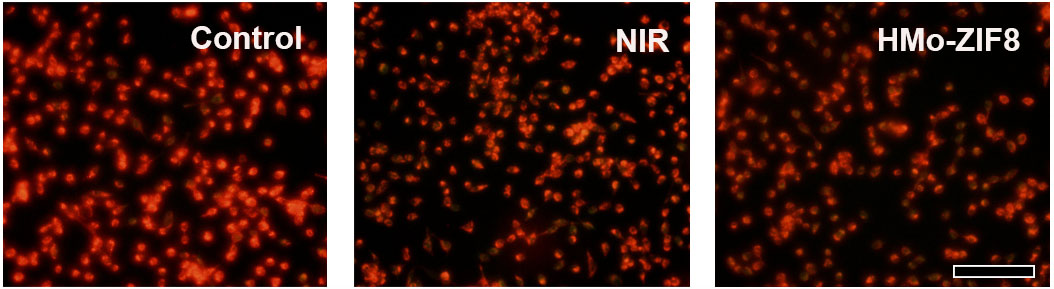
**

**Fig. S35.** CLSM images of HeLa cells stained by JC-1 after different treatments of control, NIR and HMo-ZIF8. Scale bar: 50 μm.


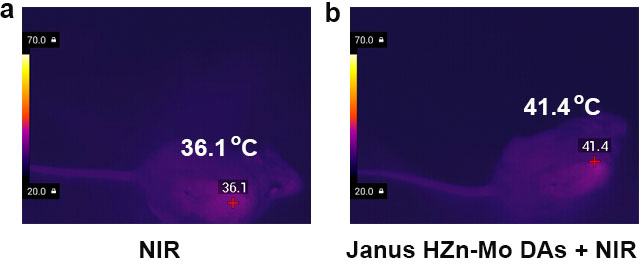


**Fig. S36.** Infrared thermal images of the representative U14 tumor-bearing mice in NIR (a) and tail vein injection Janus of HZn-Mo DAs plus NIR (b) groups after 10 min irradiation.


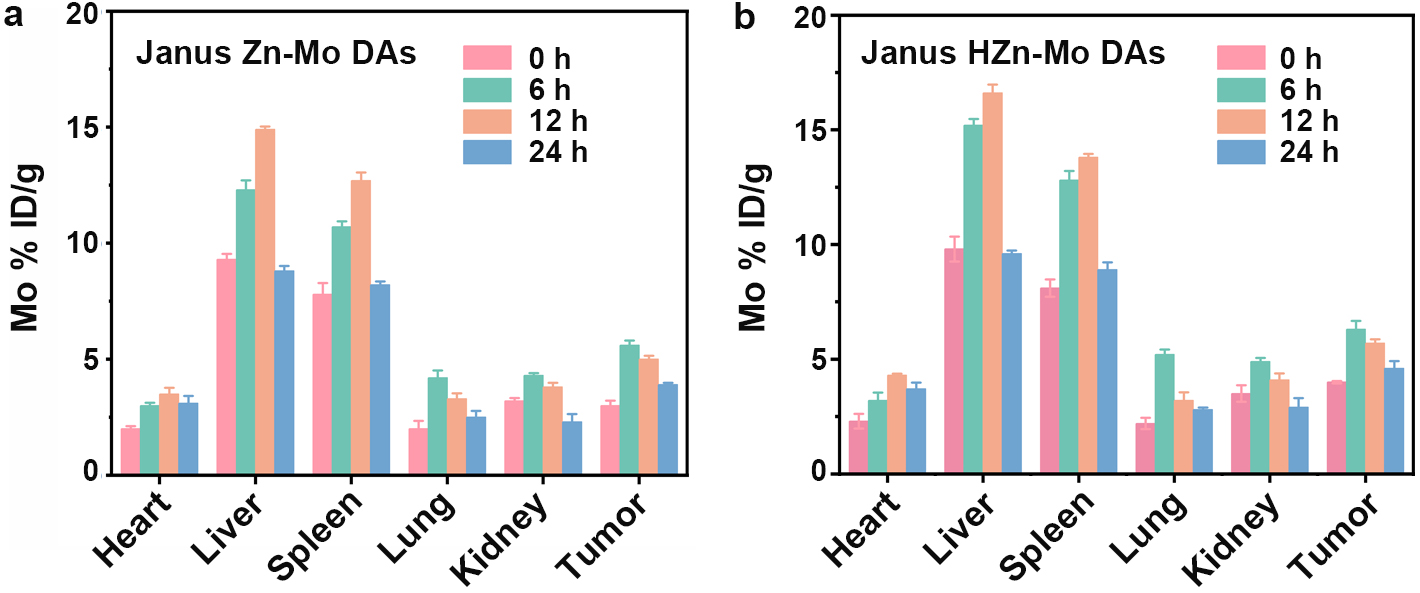


**Fig. S37.** Biodistribution Mo element in main organs (hearts, livers, spleens, lungs, and kidneys) and tumors of mice at varied time points after Janus Zn-Mo DAs (a) and Janus HZn-Mo DAs (b) administration. Data were presented as means ± SD (n = 3).


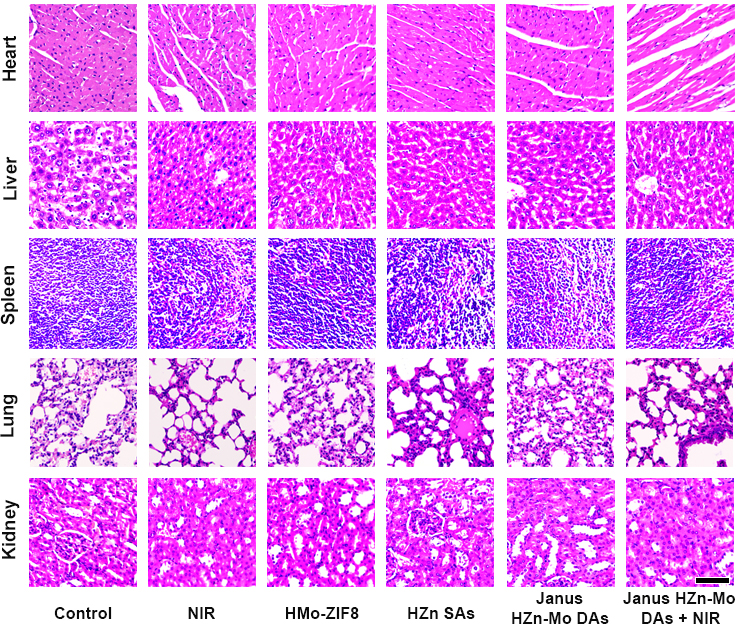


**Fig. S38.** H&E-stained tissue sections of major organs (including heart, liver, spleen, lung, and kidney) from tumor-bearing mice receiving various treatments. Scale bar: 100 μm.

**Table S1.** The contents of Zn and Mo on Janus Zn-Mo DAs tested by ICP-OES.

| **Elements** | **Zn** | **Mo** |
| --- | --- | --- |
| Wt (%) | 10.22 | 1.57 |

**Table S2**. EXAFS fitting parameters at the Zn/Mo *K*-edge for Janus Zn-Mo DAs.

| **Samples** | **Path** | ***C.N*^a^** | ***R*(Å)^b^** | ***σ*^2^×10^3^ (Å^2^)^c^** | ***ΔE_0_* (eV)^d^** | ***R* factor** |
| --- | --- | --- | --- | --- | --- | --- |
| Janus Zn-Mo DAs | Zn-N | 4.2 | 2.00 | 0.005 | 2.99 | 0.007 |
|  | Mo-O | 2 | 2.18 ± 0.01 | 0.0010 | 9.53 ± 0.45 | 0.0013 |
|  | Mo-N | 2 | 1.88 ± 0.01 | 0.0150 |  |  |

^a^*C.N*, coordination number; ^b^*R*, the distance to the neighboring atom; ^c^*σ*^2^, the Mean Square Relative Displacement (MSRD); ^d^*ΔE_0_*, inner potential correction; *R* factor indicates the goodness of the fit. Error bounds (accuracies) are estimated as follows: *CN*, ±10%; *R*, ±1%; σ^2^, ±10%.

**Table S3.** Comparison of the kinetic parameters of various nanozymes as POD-like mimetics.

| **Nanozymes** | ***K*_m_**  **(mM)** | ***V*_max_**  **(μM s^-1^)** | ***k*_cat_**  **(s^-1^)** | ***k*_cat_/*K*_m_**  **(mM^-1^ s^-1^)** |
| --- | --- | --- | --- | --- |
| Zn SAs (25 ℃) | 21.1 | 0.073 | 3.65 | 0.17 |
| Janus Zn-Mo DAs (25 ℃) | 9.6 | 0.12 | 6 | 0.62 |
| Janus Zn-Mo DAs (65 ℃) | 9.2 | 0.16 | 8 | 0.89 |

*K*_m_ is the Michaelis constant. *V*_max_ is the maximal reaction velocity. *k*_cat_ is the catalytic constant. The value of *k*_cat_/*K*_m_ represents catalytic efficiency.

**References**

[1] G. Kresse, J. Furthmüller, Efficient iterative schemes for ab initio total-energy calculations using a plane-wave basis set. Phys. Rev. B 54 (1996) 11169-11186.

[2] G. Kresse, D. Joubert, From ultrasoft pseudopotentials to the projector augmented-wave method. Phys. Rev. B 59 (1999) 1758-1775.

[3] PE. Blöchl, Projector augmented-wave method. Phys. Rev. B 50 (1994) 17953-17979.

[4] JP. Perdew, K. Burke, M. Ernzerhof, Generalized gradient approximation made simple. Phys. Rev. Lett. 77 (1996) 3865-3868.
